# Insights into Common Octopus (*Octopus vulgaris*) Ink Proteome and Bioactive Peptides Using Proteomic Approaches

**DOI:** 10.3390/md21040206

**Published:** 2023-03-24

**Authors:** Md Abdus Shukur Imran, Mónica Carrera, Sara Pérez-Polo, Jaime Pérez, Lorena Barros, Sonia Dios, Camino Gestal

**Affiliations:** Instituto de Investigaciones Marinas (IIM), CSIC. Eduardo Cabello 6, 36208 Vigo, Spain

**Keywords:** by-product, shotgun proteomics, mass spectrometry, protein-based bioinformatics, protein network, marine natural products

## Abstract

The common octopus (*Octopus vulgaris*) is nowadays the most demanded cephalopod species for human consumption. This species was also postulated for aquaculture diversification to supply its increasing demand in the market worldwide, which only relies on continuously declining field captures. In addition, they serve as model species for biomedical and behavioral studies. Body parts of marine species are usually removed before reaching the final consumer as by-products in order to improve preservation, reduce shipping weight, and increase product quality. These by-products have recently attracted increasing attention due to the discovery of several relevant bioactive compounds. Particularly, the common octopus ink has been described as having antimicrobial and antioxidant properties, among others. In this study, the advanced proteomics discipline was applied to generate a common octopus reference proteome to screen potential bioactive peptides from fishing discards and by-products such as ink. A shotgun proteomics approach by liquid chromatography coupled with tandem mass spectrometry (LC-MS/MS) using an Orbitrap Elite instrument was used to create a reference dataset from octopus ink. A total of 1432 different peptides belonging to 361 non-redundant annotated proteins were identified. The final proteome compilation was investigated by integrated in silico studies, including gene ontology (GO) term enrichment, pathways, and network studies. Different immune functioning proteins involved in the innate immune system, such as ferritin, catalase, proteasome, Cu/Zn superoxide dismutase, calreticulin, disulfide isomerase, heat shock protein, etc., were found in ink protein networks. Additionally, the potential of bioactive peptides from octopus ink was addressed. These bioactive peptides can exert beneficial health properties such as antimicrobial, antioxidant, antihypertensive, and antitumoral properties and are therefore considered lead compounds for developing pharmacological, functional foods or nutraceuticals.

## 1. Introduction

The common octopus, *Octopus vulgaris*, belongs to the coleoid cephalopods group, together with cuttlefish and squids, which are well known for their inking behavior, one of their most distinctive and defining characteristics [1]. In 1797, Cuvier first described *O. vulgaris*, which belongs to the family Octopodidae, as a benthic, neritic species that can be found in a variety of habitats, including rocks, coral reefs, and grass, from the shore to the outer edge of the continental shelf at depths ranging from 0 to 200 m [2]. Octopuses have the ability to learn, play and regenerate their damaged tissues, and they can also exhibit predatory and exploratory behavior [2,3]. In case of danger, they can squirt water at intruders to scare them away or cover themselves with ink for camouflage [4,5]. Reproductive behavior is exposed by the copulatory activity of the males; when females are ready to deposit the spawn, they hide in the dens and place the clusters of eggs on the walls [6]. Afterward, females take care of the eggs alone (the brooding period is between 25–65 days) until many of them die when the eggs hatch. They have a short life cycle of 12 to 18 months, with a rapid growth of up to 13% body weight per day with high food conversion rates of 15 to 43% that influence the culture of *O. vulgaris* [2]. Depending on the life stage, paralarvae, and settlement stage, they inhabit two different habitats. During the paralarvae stage, which lasts from hatching to around 30–40 days, animals compose part of the zooplankton and gradually transition to a benthonic life in the settlement stage [3].

As a consumer product, it is marketed both fresh and frozen and is a highly sought-after species in the market due to the qualities of its meat. A clear increase in demand and consumption of octopus can be observed in recent years, with Spain, Italy, and Japan being the main consumers and importers. Still, a rapidly growing market is opening up in the United States [7]. About 60% of the total global production of octopus comes mainly from captures in the Pacific Ocean, while the Atlantic Ocean accounts for 28% of the total production [8]. Cephalopod fisheries have grown and expanded along European coastlines due largely to the decline of traditional finfish resources, apart from the globalization of its supply and demand [9]. This increasing exploitation of cephalopods has led to a corresponding growth in the processing industries and the generation of large amounts of waste, with considerable disposal costs.

The processing of squids, cuttlefish, and octopus generates large amounts of solid and liquid wastes in the forms of skin, head, cuttlebone, pen, ink, and viscera [10]. However, octopus ink by-products have received much attention due to the discovery of several relevant bioactive compounds [1,10,11,12].

Cephalopod ink is mainly composed of a mixture of secretions from the ink gland and the mucus-producing gland. The ink gland primarily belongs to the ink sac that produces black melanin-based ink and releases its secretion into the ink sac lumen, before being discharged into the hindgut near the anus via a duct. The funnel organ, the only mucus gland close to the ink sac, secretes and releases mucus simultaneously with the ink sac’s secretion [1]. Melanin is the most important natural pigment present in cephalopod ink, which is a complex biopolymer that usually exists in two forms, eumelanin and pheomelanin [1]. It is produced from the amino acid tyrosine by a series of biochemical conversions and constitutes ~15% of the total wet weight of ink [1,13,14]. Proteins make up another 5–8% of the weight of cephalopod ink [15]. Additionally, several peptidoglycans-polysaccharides and oligopeptides have been isolated from the ink of several squid species [1,16,17,18]. These pigments, amines, chemicals, proteins, peptidoglycans, or polysaccharides could be valuable sources of bioactive substances.

Marine species are abundant sources of bioactive substances with a wide range of structural variations and biological activity and are becoming increasingly significant as a source of novel bioactive compounds [19]. These peptides are reportedly found in sponges, ascidians, seaweed, and mollusks and exhibit a variety of pharmacological characteristics [20,21]. Bioactive peptides have been identified as certain protein fragments that have a favorable effect on physiological conditions or processes and may have a health-promoting effect [22]. During gastrointestinal digestion, these peptides can be released from their precursor proteins or produced by in vitro proteolytic processes with exogenous proteases. Bioactive peptides and their precursor proteins are crucial for living organisms’ metabolic functions and human health. Based on their mode of action, they can be categorized as antibacterial, antithrombotic, antihypertensive, opioid, immunomodulatory, mineral binding, and antioxidative. They also exhibit hormone-like or drug-like properties [22,23]. In this sense, bioactive peptides are lead compounds for developing pharmacological, functional foods or nutraceuticals.

The main aim of this study was to apply the advanced proteomics discipline to generate an octopus ink reference proteome for the screening of potential bioactive peptides from fishing and aquaculture discards and by-products.

## 2. Results

### 2.1. Octopus Ink Samples

Due to varying sampling methods and ink sac sizes, we collected different amounts of ink samples, which directly influenced the recovery of ink proteins. Table 1 summarizes all the sample collection methods and the recovery of ink proteins.

**Figure 1:**
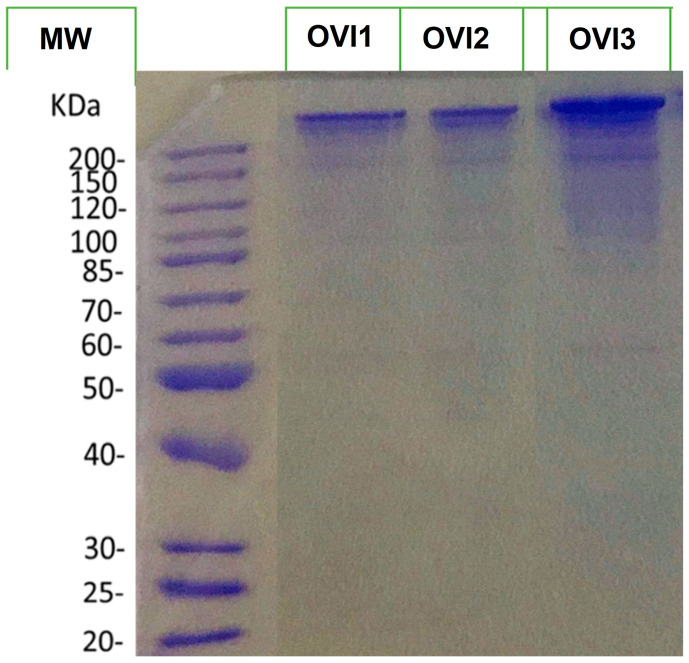
SDS-PAGE 10% profiles of the extracted proteins of *O. vulgaris* ink samples. MW denotes molecular weight.

Additionally, to visualize the protein profile of the octopus ink samples, the three biological ink samples were separated by SDS-PAGE 10% (Figure 1). This gel illustrates that all extracts show a similar protein profile.

### 2.2. Octopus Ink Proteome

A reference octopus ink proteome was created by merging 2693 identified spectra (Peptide Spectrum Matches, PSMs) from 1432 different peptides obtained from three different octopus ink samples (Supplementary File S1). Finally, a total of 361 non-redundant annotated proteins were identified from these peptides (Supplementary File S2). This discovery stage was based on the LC-MS/MS analysis and SEQUEST-HT search of the tryptic digestion for the total protein extracts from each octopus ink sample compiled in a unique dataset. Raw data and analyses outputs are publicly available in the MassIVE data repository under accession number MSV000089896 and the ProteomeXchange database under accession number PXD035359.

One of the major limitations to work with non-model organisms is the scarcity of public protein and gene databases. This is the reason why protein identification was conducted using Proteome Discoverer 2.4 (Thermo Fisher Scientific, San Jose, CA, USA), using both a global database according to phylogenetic similarity for the class “Cephalopoda” with about 125,800 protein entries, including canonical and isoforms sequences in UniProtKB protein database, and also the UniGene transcriptomic database of octopus paralarvae traduced to proteins [3], containing 77,838 protein sequences, which considerably increased the number of protein identifications. Supplementary File S2 summarizes the proteins followed by their respective gene name, gene homologs, PMS, unique peptides, and percentage of protein coverage. Of the 361 proteins detected, a total of 208 proteins were assigned to the species *O. vulgaris*. A total of 37 uncharacterized proteins were observed, 17 of them related to *O. vulgaris*.

The final global dataset of the octopus ink proteome was subsequently analyzed by protein-based bioinformatics, such as gene ontologies, pathways, and network analyses, and by the prediction of potential bioactive peptides to gather more functional insights of the octopus ink.

### 2.3. Label-Free Quantification (LFQ) of O. vulgaris Ink Samples

**Figure 2:**
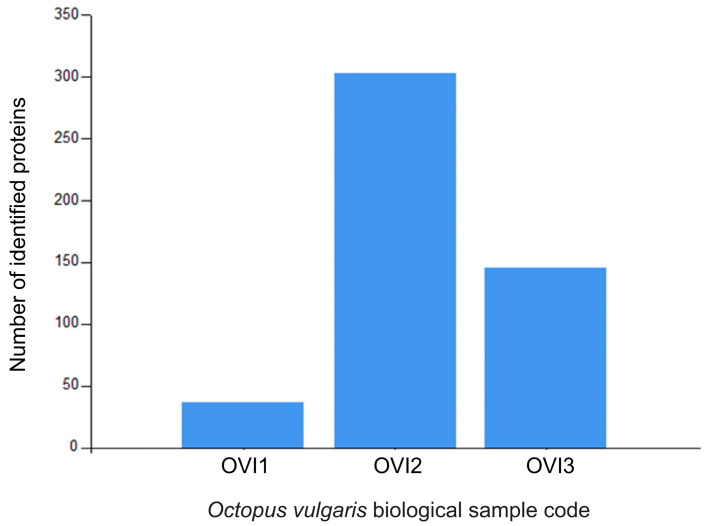
Distribution of the high-abundance proteins for *O. vulgaris* ink samples (OVI1, OVI2, OVI3) determined by LFQ.

**Figure 3:**
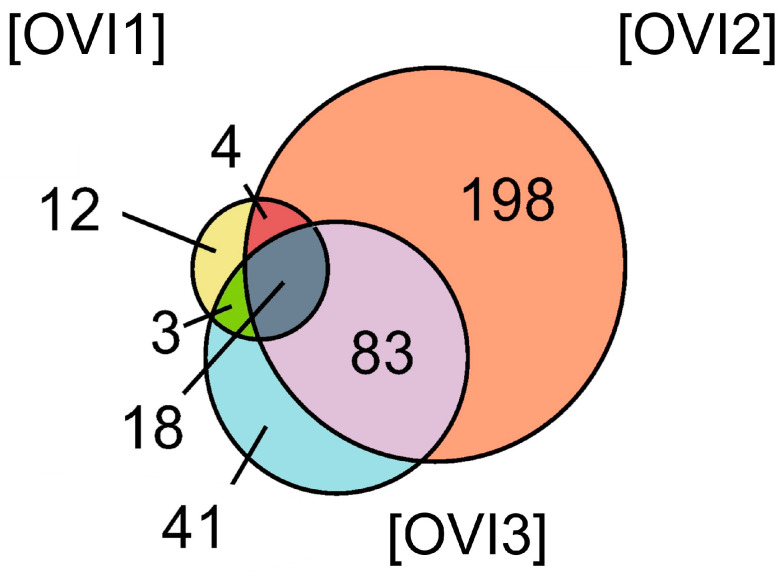
Venn diagram of the high-abundance proteins for all the *O. vulgaris* ink samples (OVI1, OVI2, OVI3) determined by LFQ.

Relative label-free quantification of each *O. vulgaris* ink sample (OVI1, OVI2, OVI3) was also performed to determine the protein abundance of each sample. Supplementary File S1 contains these results. High-abundance proteins for each sample were analyzed and compared. Figure 2 shows the distribution of high-abundance proteins detected for each *O. vulgaris* ink sample, while Figure 3 (Venn diagram) shows the distribution and overlapping of high-abundance proteins for all *O. vulgaris* ink samples. The determination of proteins in these samples was directly influenced by ink sac sizes, sampling techniques, and protein precipitation. As was demonstrated in Figure 2 and Figure 3, the majority of the high-abundance proteins were detected in the OVI2 sample. In our study, the syringe method (OVI1) retrieved the fewest proteins, but the milking method after euthanasia generated the opposite outcome (Table 1, Figure 2 and Figure 3). Moreover, protein precipitation resulted in a minor loss of proteins for OVI1 and OVI3, whereas no purification led to the identification of significant protein abundance for OVI2. These hybrid collection methods offer a high coverage of the octopus ink proteome.

### 2.4. Functional Analysis: Gene Ontologies and Pathways Analysis

**Figure 4:**
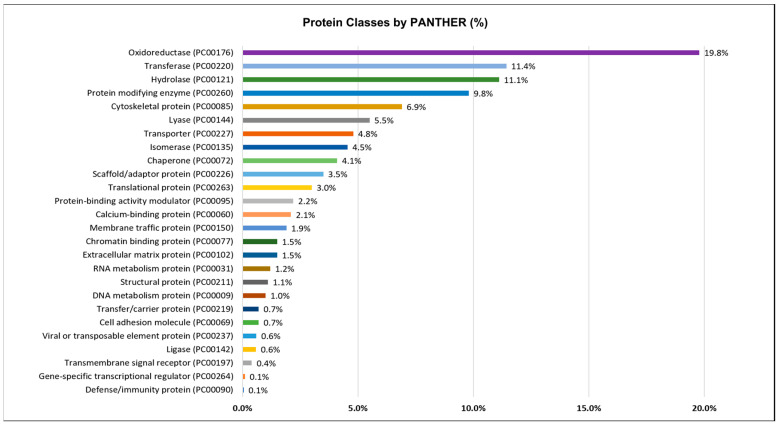
Protein classes of the octopus ink proteome identified by shotgun proteomics and categorized by PANTHER (http://pantherdb.org/, accessed on 3 June 2022) using the homologous gene list.

PANTHER analysis of the octopus ink proteome using homologous genes (*O. bimaculoides; Homo sapiens; Drosophila melanogaster*) of *O. vulgaris* revealed the presence of 26 different protein classes (Figure 4). Apart from protein class identification, PANTHER was used to categorize the ink proteomes based on their molecular function and biological process (Supplementary Figures S1 and S2). For the prediction of different proteins classes and function, PANTHER analysis used a number of genes, a percentage of genes, and functional hits against total genes. All of these corresponding data up to gene level 2 were compiled on Supplementary File S3.

Figure 4 shows that oxidoreductase (19.8%), transferase (11.4%), hydrolase (11.1%), protein modifying enzyme (9.8%), or cytoskeletal protein (6.9%) were the most prominent protein classes. Protease (7.45%) was the most common protein modifying enzyme, followed by protein phosphatase, tyrosine protein kinase, serine/threonine protein kinase, and ubiquitin-protein ligase (Supplementary File S3).

A significant part of the ink proteins involved in catalytic activity (GO:0003824) and binding (GO:0005488) were revealed through molecular function analysis (Supplementary Figure S1) where hydrolase (20.91%) and oxidoreductase activity (15.82%) were the most common catalytic activities (Supplementary File S3). Other molecular function activities of ink proteins such as transporter activity (GO:0005215), molecular function regulator (GO:0098772), ATP-dependent activity (GO:0140657), and structural molecule activity (GO:0005198) were also found.

Moreover, ink proteome analysis by PANTHER identified proteins implicated in 16 different biological processes (Supplementary Figure S2). Most of the proteins were involved in the cellular and metabolic process followed by a response to stimulus, biological regulation, localization, and signaling. It also revealed that the octopus ink proteome was involved in the immune system process by performing similar activities such as leukocyte activation (GO:0045321), leukocyte migration (GO:0050900), immune system development (GO:0002520), immune effector process (GO:0002252), and immune response (GO:0006955) (Supplementary File S3). Among these processes, leukocyte-like activation (32.60%), immune response (25.80%), and effector (17.20%) were the most prominent activities.

Additionally, the PANTHER pathway analysis of homologous genes of common octopus ink proteomes identified 70 different pathways based on their functional hits (Supplementary File S3). Among all of these pathways, angiogenesis (P00005), apoptosis signaling pathway (P00006), toll receptor signaling pathway (P00054), serine glycine biosynthesis (P02776), FGF signaling pathway (P00021), and EGF receptor signaling pathway (P00018) were highlighted as some important immune signaling pathways in the octopus ink proteome.

The KEGG Pathway systematic analysis of identified proteins was carried out by the DAVID program (version 6.8) to compare the input data with the background of the *O. bimaculoides* genome, which is the most phylogenetically closest cephalopod species available in the DAVID software. The KEGG pathway search identified 21 different pathways, and the majority of the proteins were found to be involved in metabolic pathways, amino acid biosynthesis, or xenobiotics and drug metabolism (Supplementary File S4).

DAVID software was also used to identify the functional domain of ink proteins. In this case, the InterPro motifs platform was selected for domain searching, categorizing a list of proteins based on protein functional domain (Supplementary File S5).

### 2.5. Network Analysis

A comprehensive protein network encompassing both functional and physical protein interactions was constructed by combining all of the proteins identified for the octopus ink proteome using the STRING software version 11.5. As the genome of *O. vulgaris* is not available in the STRING software, *Octopus* spp. was selected, providing a protein–protein interaction (PPI) enrichment *p*-value of less than 1.0 × 10^−16^, and a total of 147 nodes (proteins) and 277 interactions (edges) were discovered.

**Figure 5:**
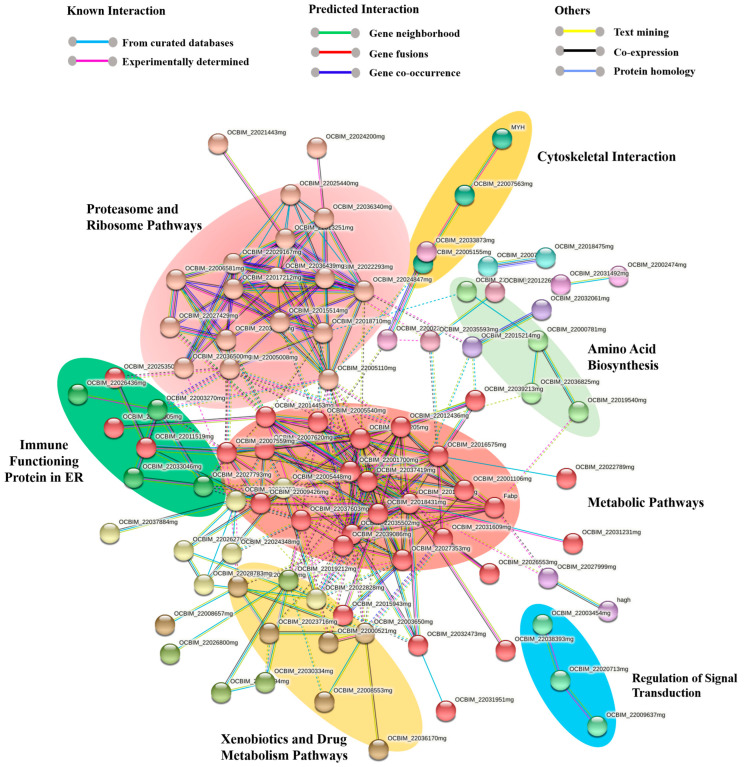
Protein network for the common octopus ink proteome using the STRING software (v.11.5). Proteins are represented with nodes. Physical direct interactions are represented with continuous lines and functional indirect interactions with dotted lines.

A total of 15 subgroups were obtained from 147 nodes, where all the disconnected nodes were hidden from the final network (Figure 5; Supplementary File S6). Among these subgroups, all the significant pathways with at least three nodes were highlighted in Figure 5. In the octopus ink proteome, metabolic pathways (red) with 30 nodes and 99 interactions made up one of the major pathways, along with ribosome and proteasome pathways with 66 protein–protein interactions (salmon pink; nodes: 18). Different immune proteins part of the innate immune system such as ferritin, catalase, proteasome, and Cu/Zn superoxide dismutase were found in these pathways.

Subgroups of xenobiotics and drug metabolism by the cytochrome P45 (gold; 7 nodes) and immune functioning proteins in the endoplasmic reticulum (dark green; 4 nodes) such as calreticulin, disulfide isomerase family, and heat shock protein 70 family were also identified in the ink proteome. Cytoskeletal protein interactions (yellow; 3 nodes) were primarily formed by the actin and myosin proteins, while signal transduction regulation was mediated by small GTPase (blue; 3 nodes). All the proteins involved in these processes were also identified through molecular functional studies (Supplementary Files S3–S5).

### 2.6. Putative Bioactive Peptides

In this study, an octopus ink proteome (n = 361) was used to predict all the converted bioactive peptides. For the prediction of the active peptide sequence, protein hydrolysates with trypsin and pepsin were performed using the MS-Digest computational program. No missed cleavages and a minimum of six residues per peptide were selected as parameters. All the predicted peptides after every enzymatic digestion (pepsin and trypsin) are presented in Supplementary File S7.

Trypsin-digested peptides were evaluated for potential bioactivity through Peptide-Ranker (PR), releasing more than 10,000 different peptides (6–44 amino acid residues). A total of 111 non-redundant peptides were selected, which scored higher than 0.90 using the N-to-1 neural network probability (Table 2). The majority of the bioactive peptides from tryptic digestion corresponded to prominin, tetraspanin, hemocyanin, peroxidases, mucin, and some uncharacterized proteins, for which most of them belong to *O. vulgaris* and *O. bimaculoides*.

Similarly, the second in silico digestion of octopus ink proteins with pepsin yielded more than 7000 peptides, with 6 to 44 amino acid residues (Supplementary File S7). Among them, 15 bioactive peptides (score > 0.9) were identified by the Peptide-Ranker with their parent protein (Table 3). Pepsin-digested bioactive peptides mostly belong to prominin, retinal dehydrogenase, hemocyanin subunit, and heat shock proteins.

All the bioactive peptides from trypsin and pepsin digestion (n = 126) were further evaluated for their antimicrobial potential using CAMPR3 (Collection of Antimicrobial Peptides) integrated in the BIOPEP-UWM database. In addition, properties of peptides, e.g., allergenicity and toxicity, were also evaluated by widely used computational platforms AllerTop and ToxinPred (Table 2 and Table 3, respectively). The majority of bioactive peptides from trypsin digestion (n = 111) belong to the non-allergen and non-toxin peptides group. Among them, 39 peptides showed antimicrobial potentiality, where 10 peptides scored more than 0.90 in the discriminate analysis classifier score. Additionally, 15 antimicrobial peptides (AMPs) from tryptic digestion showed both non-allergen and non-toxin reactivity. These peptides included mucin-5ac-like (MUC5AC), filamin-a, hemocyanin, inter-alpha-trypsin inhibitor heavy chain, s-formylglutathione hydrolase, tetraspanin, glyoxylate reductase, DNAH, prostaglandin reductase, myosin heavy chain (MYH), H(+) transporting two-sector ATPase, thyroglobulin, and S (hydroxymethyl)glutathione dehydrogenase proteins.

In the case of pepsin digestion, a total of 10 peptides showed potential antimicrobial capability with a higher discriminate analysis classifier score. Among these antimicrobial peptides, the majority of peptides showed both non-allergen and non-toxin reactivity through computational analysis. These hydrolysates were part of prominin, hemocyanin, heat shock protein, retinal dehydrogenase, or acid ceramidase.

## 3. Discussion

In this study, a common octopus, *O. vulgaris,* ink proteome was generated for the first time by using shotgun proteomics, and a total of 361 non-redundant proteins were identified from the complex mixture of ink samples. A shotgun bottom-up proteomics approach is a widely used protocol to create a reference dataset of proteomes for selected marine by-products whereby enzymatically digested peptides from complex samples are used to identify proteins [24,25].

Extracted protein samples for OVI1 and OVI3 were discovered to have a blackish color that could interfere with concentration measurements. Thus, protein samples were further purified and quantified. We found that there was a small loss of proteins during protein precipitation, but the presence of a large amount of proteins was detected in OVI2 that had not been purified. Moreover, a number of protein identifications also varied depending on the extraction technique used. Prior research has demonstrated that the syringe or milking technique has an effect on the recovery of chemical components and proteins from cephalopod ink [1,26]. Hence, the final merging of these samples offers a more precise depiction of the ink proteome. Octopus ink proteome is available at a public repository and could be exceedingly advantageous for future marine by-product research and industrial applications.

Subsequent computational analysis through PANTHER identified 26 active protein classes in the octopus ink proteome. Among these classes, oxidoreductase was the most relevant protein class. In octopus ink, oxidoreductases are primarily involved in the melanogenesis process for catalyzing the polymerization of eumelanin and in the antimicrobial defense system [1,27]. A peroxidase enzyme found in the cephalopod ink sac and associated with melanin synthesis process [28,29] was also recovered from the octopus ink proteome in a large proportion. Additionally, the percentage of protein coverage for peroxidase was high, with excellent peptide spectrum matching, as it was also for hemocyanin and CD109 antigen. The enzymes hemocyanin, tyrosinase, and phenoloxidase share similar active sites. Molluscan hemocyanins are responsible for both oxygen transfer and an effective innate immunological response, while tyrosinases start the synthesis of melanin [30,31,32]. Phenoloxidase also plays an important role in the initial immune defense of invertebrates as a part of the prophenoloxidase-activated system [33]. Fan et al. purified phenoloxidase from ink sacs of *O. ocellatus*, which is involved in melanin production as well as in-host defense via melaninization as in other crustaceans [34]. Cephalopod ink is composed of secretions from two glands, the ink gland and the funnel organ, a mucus-producing gland, both irrigated by blood vessels. The presence of hemocyanin (a protein that transports oxygen, which is synthetized in cephalopods mainly in the branchial hearts and released to the bloodstream) could be attributed to discharges or the rupturing of vessels in the ink sac, but it could be also a proper component of the ink. Since no previous studies at the proteomic level have been performed before in common octopus ink, and taking into account that hemocyanin has been identified in different organs, including mucus coating different epithelia, it could be possible that hemocyanin, as other phenoloxidases, could be part of the mucus secreted by the ink gland or funnel organ. Further studies are needed to clarify this aspect. Cell surface antigen CD109 is a member of thioester-containing proteins, which form part of the innate immune system involved in host–microbe interactions that have been reported to recognize and bind, and phagocytose bacteria and other parasites [35,36,37].

The KEGG pathway and network analysis of the octopus ink proteome by DAVID (v-6.8) identified 21 different biological pathways, where most of the proteins were involved in metabolic pathways, amino acid biosynthesis, or xenobiotics and drug metabolism. Similar functional and physical protein interactions for all identified proteins of ink were found by STRING. A total of 147 proteins and 277 interactions were discovered through interaction analysis, which covered all the KEGG pathways identified. MCL cluster analysis categorized 15 subgroups from 147 nodes, where metabolic pathways (red; nodes: 30), ribosome and proteasome pathways (salmon pink; nodes: 18), and xenobiotics and drug metabolism by cytochrome P45 (gold; nodes: 7) were identified as the major pathways. Glycolysis, the TCA cycle, the biosynthesis of nucleotide sugars, oxocarboxylic acid metabolism, and oxidative phosphorylation were the major metabolic pathways, and comparable results were obtained from previous ink gene ontology studies using DAVID and PANTHER. These metabolic networks were also identified through transcriptomics analysis in some previous studies [38,39,40]. Another significant iron soluble non-toxic protein ferritin was found in the metabolic networks of octopus ink, which is involved in the immune system and homeostasis process [41]. Catalase, also found in the ink metabolic network, scavenges free radicals to curtail their damaging effects on the host, and it is a crucial enzyme in antioxidant defense and the innate immune system [42].

Moreover, the ubiquitin-proteasome pathway is directly involved in cellular apoptosis, and in some cases, the proteasome can impact other cellular pathways, which may lead to apoptosis [43]. In this study, ubiquitin-activating enzyme E1, E3 ubiquitin-protein ligase, 26S proteasome subunit, proteasome subunit alpha/beta, and proteasome A-type subunit were identified from the common octopus ink. We also identified cytochromes P450 (CYPs), which are a superfamily of enzymes catalyzing xenobiotics in marine invertebrates [44]. All of these identified pathway and immune molecule activities have been described as an essential part of the common cephalopod immune system [45,46]. Another important immune protein, Cu/Zn superoxide dismutase, which is a part of antioxidant defense pathways, clusters in the ink proteasome and ribosome pathways [47,48].

Four immune functioning proteins in the endoplasmic reticulum such as calreticulin, the disulfide isomerase family, the heat shock protein 70 family, and carboxypeptidase were identified from the ink proteome. Calreticulin, a highly conserved endoplasmic reticulum (ER) luminal resident protein, is involved in innate immunity and Ca2+ homeostasis [49]. Huang et al. reported that two ER proteins, calnexin and calreticulin, were involved in antibacterial immunity in *Eriocheir sinensis* [50]. The disulfide isomerase family functions as molecular chaperones and disulfide oxidoreductase. Through a variety of cellular processes, including redox-sensitive attachment, antigen presentation in the ER, connection with phagosomes, and ROS production by NADPH oxidase, protein disulfide isomerase promoted host–pathogen interactions in viral, bacterial, and parasitic infections [51]. By using cDNA cloning and mRNA expression of heat shock protein 70 (HSP70) gene, Song et al. showed that HSP70 plays a key role in mediating the environmental stress and immune response in bay scallops [52]. Carboxypeptidase, which belongs to the S10 peptidase family, was also purified from *Illex illecebrosus* [53,54].

Bioactive peptides have been defined as specific protein fragments that have a positive impact on body functions or conditions and may ultimately influence health. Peptides are inactive within the sequence of the parent protein but become active when released due to the action of different enzymes [55,56]. Peptides from different cephalopod extracts showed antibacterial activities, which is an important function of the innate immune system. Positively charged amino acids of peptides interact with the negatively charged membranes of microorganisms to permeate the cell and finally exert their antimicrobial effects [57]. Cephalopod ink is widely used in traditional Chinese medicine due to its antitumor, immunomodulatory, and hemostatic effects [58]. In addition, cephalopod ink secondary metabolites promoting immune function in vertebrates also showed different bioactive potentials, such as antibacterial, antimutagenic, and antitumoral activity [10]. Other studies evidenced that octopus ink extracts exhibited joint immunomodulatory and antiproliferative effects due to the presence of different bioactive compounds without being cytotoxic to human cancer cell lines [12]. Limited data are available regarding the activity of the bioactive peptides found in *O. vulgaris* ink; thus, this study may offer a new approach to identifying potential lead bioactive peptides from *O. vulgaris* ink.

Trypsin and pepsin in silico enzymatic digestions from the LC-MS/MS reference octopus ink proteome identified more than 17,000 peptides. Trypsin preferentially cleaves the proteins at Lys and Arg residues in position P1, except for the case in which Pro is found in position P1′, where pepsin cleaves the proteins at Phe, Tyr, Trp, and Leu residues in positions P1 and P1′ [59]. Previous studies showed that the trypsin fractioning of isolated peptidoglycans of *S. maindroni* ink released a polysaccharide with strong antimutagenic activity [58] and that trypsin was also used to hydrolyze oligopeptides to produce a proapoptotic tripeptide [18]. Similarly, trypsin, α-chymotrypsin, or pepsin hydrolysates of giant squid tunic gelatin exhibited antioxidant activity [60].

The bioactivity of peptides was predicted by PeptideRanker, which calculates scores ranging from 0 to 1, assigning higher values to those peptides considered more bioactive [61]. A total of 111 non-redundant bioactive peptides from tryptic digestion and 15 bioactive peptides from pepsin digestion were selected using PeptideRanker. The proteins hemocyanin, prominin-1-a isoform, retinal dehydrogenase, and acid ceramidase released bioactive peptides after in silico digestion with both trypsin and pepsin enzymes.

Hemocyanins are invertebrate metalloproteins found in cephalopods and are mainly known for their role in oxygen transport. Coates and Nairn mentioned that hemocyanins act as a precursor of antimicrobial and antiviral peptides [62]. These proteins play important immune-related roles, such as antimicrobial, antiviral, agglutinative, antifungal, and antitumor proliferation of cancer cells [63,64,65]. In fact, the hemocyanin of marine mollusks has shown significant interactions with T cell monocytes, macrophages, and polymorphonuclear lymphocytes to improve the host immune response [66,67]. Although no previous studies are available related to octopus hemocyanin, it can be considered that the potential pepsin- and trypsin-digested bioactive peptides (SDPMRPF, CGVCPKCHF, IPCLFAIVFAFWLCGHIAEGNLIR, KKPMMPF, VFGGFWLHGIK, MFAGFLLK, SPWLLGATILCIISIFVPVITNGK, ILCLFAFVFAFWLSGQSAEGNLIR, YACCLHGMPVFPHWHR, VFAGFLFMGIK, VFVGFLLHGFGSSAYATFDICNDAGECR, LNHLPLLCLAVILTLWMSGSNTVNGNLVR, TSFLFLAFVATSWFVYAVTASK) from hemocyanin proteins could be used in the future in antimicrobial, antiviral, anticancer or potential immune stimulator roles.

Prominin-1 is a membrane glycoprotein specifically associated with plasma membrane protrusions, first identified as a novel antigenic marker that is present at the apical surface of mouse neuroepithelial cells [68,69]. It is a very useful marker for various stem cells and can be found in a wide variety of differentiated epithelium and non-epithelial cell types, including photoreceptor cells of invertebrate where mutations in the PROM1 gene are associated with various forms of retinal degeneration [70,71]. Genome sequencing revealed that prominin relatives are present in different echinoderms and mollusks where the amino acid sequence is poorly conserved among prominin-1 gene products [71,72]. In addition, the extensive research of human PROMININ-1 as a possible target for cancer treatment in various organs was realized [71]. Therefore, the discovery of prominin-1-a isoform from octopus ink as well as bioactive peptides (CCCRCCNRCGGRHMKY, IVLYFIGYSICVAIGILFIILIPLIGCCLCCCR, TYVTCLVILNTIILFAVVCTFITNELYK, SVAVPCSVLLLWILIAFSLVDHSFAQNSSQQHR) from this protein may open up new opportunities for cancer and stem cell studies.

Retinal dehydrogenase belongs to the super family of aldehyde dehydrogenases and catalyzes the chemical reaction converting retinal to retinoic acid [73]. Aldehydes are highly reactive molecules that may produce carcinogenic, cytotoxic, mutagenic, and genotoxic effects on biological systems where aldehyde dehydrogenases transform aldehydes to less reactive forms or eliminate the aldehydes [74]. Bioactive peptides (CMGQCCF, IMTFTNAIQAGTVWVNTYCCVACQAPFGGFK) released from retinal dehydrogenase could be useful to minimize the toxic effect of cancer cells and for future cancer research.

The sphingolipid enzyme acid ceramidase has played an important role in the regulation of apoptosis and also was found to be over-expressed in different human cancer cells [75,76]. Acid ceramidase, which is involved in the initiation and propagation of a number of human cancers could act as a potential therapeutic target in cancer therapy [76]. However, an acid ceramidase-like protein was identified from the ink proteome, whose function is still unknown in cephalopods to the best of our knowledge. In addition, bioactive peptides released from this protein have not shown any antimicrobial potentiality through in silico analysis.

In the present work, antimicrobial peptides (AMPs) were identified using the CAMP (Collection of Anti-Microbial Peptides) database and by applying the DAC score (Discriminate Analysis Classifier score), since CAMPR3 is a widely used database for the prediction of antimicrobial peptides [77]. A total of 39 tryptic peptides showed antimicrobial potential, while 10 peptides showed potential antimicrobial capability for pepsin digestion. The majority of the tryptic-digested antimicrobial peptides were released from hemocyanin, tetraspanin, peroxidase-like protein, myosin heavy chain, MUC5AC, filamin-a, inter-alpha-trypsin inhibitor heavy chain, and s-xymethyl glutathione dehydrogenase, among others. Heat shock protein is another important protein that released antimicrobial bioactive peptides due to pepsin digestion.

The myofibrillar protein myosin heavy chain, one of the key elements of the muscle, plays a role in both muscular contraction and non-muscular cells, which was previously found in the octopus arm using proteolytic assay [78]. MYH released bioactive peptides (NWQWWR) with high AMP probability, which could be used in future antimicrobial research.

MUC5AC, a major gel-forming mucin, exerts a protective role against inhaled pathogens, while some other studies described that mucin proteins act as a barrier to different microorganisms functioning in a dynamic role in host innate and adaptive immune responses to infection [79,80]. Previous studies showed that mucin-5ac-like proteins have been identified from ivory shell haemocytes of *Babylonia areolata* significantly involved in the immunological homeostasis of invertebrates [81]. Intestinal mucin isolated from *Trichoplusia ni* facilitates the digestive process and protects invertebrate digestive tracts from microbial infections [82]. Thus, we could predict that the identified mucin-5ac-like protein and bioactive peptides (SSFDGGSFGGGIAAGIAIAILLLALIYLFYR) from the octopus ink might be useful for future antimicrobial research and applications.

Tetraspanins, which were identified in octopus ink proteome-releasing bioactive peptides, are a group of four-transmembrane domain proteins involved in cell–cell adhesion at cellular junctions or bacterial cell adhesion [83]. Some tetraspanins are capable of limiting cancer progression or migrations, while others foster tumor growth, invasion, and metastasis [84]. Future extensive research is needed to determine the tetraspanin role in *O. vulgaris* and the potential bioactivity of its peptides (IAAAGLALAFIQVIGIVFACCLAQAIR).

Heat shock proteins act as molecular chaperones in the immunity of organisms, especially under different environmental stresses [85,86]. The inking behavior of cephalopods increased HSP90 expression and suggested that the stimulated increase in HSP90 expression level was one of the organisms’ protective approaches against further toxicity [87]. Previous studies revealed that HSP70 releases bioactive peptides and exhibits different biological activities, including ACE inhibition, antioxidant, dipeptidyl peptidase-IV inhibition, etc., through proteomic and bioinformatic analysis [88]. Therefore, pepsin-digested bioactive peptide (GGMPGGMPGGMPGGMPNF) from heat shock proteins could be a new area of natural product research interests.

Additionally, peptide properties were evaluated through AllerTop and ToxinPred web servers. AllerTop predicts the allergen peptides based on the physicochemical properties of peptide sequences [89]. Similarly, ToxinPred compiled different toxic and non-toxic peptides from the known database of SwissProt and TrEMBL and developed in silico models for the toxicity prediction of peptides and proteins [90]. Most of the antimicrobial peptides of ink proteins released from pepsin and trypsin digestion showed non-allergen and non-toxic reactivity properties thorough in silico analysis.

Some of the previous studies reported that cephalopod ink extracts possess antimicrobial properties against diverse pathogenic bacteria [1,91]. Additionally, recently, it was found that *O. vulgaris* ink extracts exhibit anti-inflammatory, antiproliferative, antimutagenic, antioxidant, and cytoprotective properties [11,12]. There is currently no information concerning cephalopod ink bioactive peptides, but Nadarajah et al. mentioned that fractions of melanin-free ink with low molecular weights (<3 kDa) showed the highest antioxidative activities [92]. Thus, low molecular weight peptides of octopus ink identified for the first time in the current study could be a potential resource in future antimicrobial, antioxidant, and anticancer research. These potential bioactive peptides must be validated by further functional analysis utilizing synthetic peptides to confirm the bioactivity of these potential candidates. In this sense, the bioinformatics method offers quicker and less expensive alternatives than the conventional methods to reduce the number of potential targets that need to be explored.

## 4. Materials and Methods

### 4.1. Common Octopus Sampling

A total of three octopuses (*O. vulgaris*) with an average weight of 1 kg (980 g to 1.2 kg) were collected using fishing cages by professional certificated fishermen at the Ría de Vigo, Spain. The individuals were transported in proper containers to the Experimental Culture Facilities of IIM-CSIC, which is registered as “User and breeding center on animal experimentation” ES360570202001. Transport, housing, and handling were carried out following the principles of animal welfare, within 2 h after fishing. Special attention was paid to the 3Rs strategy (Reduce, Refine, Reuse), reducing the number of animals used in the experimental assay until it was essential for maintaining statistical robustness. For ink extraction, an initial less invasive method was tested using a live, anesthetized octopus with an anesthetic mix of MgCl_2_ (1.5%; *w/v*) and 70% ethanol (1%; *v/v*) dissolved in sea water [93]. Ink was then extracted in vivo using a syringe. Since this method produced very small amounts of ink, octopuses were euthanized using overdoses of the anesthetic (MgCl_2_ (3%; *w/v*) and 70% ethanol (1%; *v/v*) dissolved in sea water) and were carefully dissected using sterilized scissors. Ink sacs were collected by the “milking” method (the content of the ink sac was milked by running forceps along its length) from two octopuses, and ink was transferred into a collection tube. Finally, a total of three representative ink samples (OVI1, OVI2, OVI3; OVI: *O. vulgaris* ink) were selected for further analysis and were stored at −80 °C.

Procedures for transportation, maintenance, euthanasia, and dissection were carried out in accordance with the principles published in the European Directive (2010/63/EU) for the protection of experimental animals used for scientific purposes and were approved by the Spanish National Competent Authority ethics committee (Research Project ES360570202001/17/EDUCFORM 07/CGM02).

### 4.2. Ink Protein Samples

All the samples were homogenized on ice for 6 cycles of 5 s pulses in an ultra-turrax (Polytron Aggregate^R^, Kinematica-AG, Switzerland) using 4 mL of lysis buffer (10 mM Tris–HCl, pH 7.2 with 5 mM phenylmethylsulfonyl fluoride (PMSF)), which was prepared fresh [56]. Homogenized ink samples were centrifuged at 40,000× *g* for 20 min at 4 °C in an Avanti JXN-26 centrifuge (Beckman Coulter, Palo Alto, CA, USA). Extracted protein samples were purified to remove the blackish ink color using the methanol-chloroform precipitation method [94] to avoid interference with further protocols. The OVI2 sample was analyzed without purification due to the recovery of the almost transparent protein extract (light blackish color). Protein concentration in each protein extract was measured by a BCA (Bicinchoninic acid) protein assay kit (Pierce^TM^-23225) in a spectrophotometric device (Multiskan™ GO, Thermo Fisher Scientific). Extracted proteins were stored at −80 °C until use.

### 4.3. SDS-Polyacrylamide Gel Electrophoresis

Octopus ink proteins of each individual sample were separated onto 10% (*v/v*) of SDS-polyacrylamide gels (30% acrylamide/N, N’-ethylene-bis-acrylamide, 37.5:1) with a stacking gel of the same polyacrylamide concentration. A total of 15 µg of proteins with an equal amount of Laemmli buffer (Tris-HCl 0.5 M pH 6.8; Glycerol; SDS 10%; Blue Bromophenol 1%; DTT) was boiled on a thermocycler at 100 °C for 5 min and centrifuged at 10,000× *g* 2 min at 4 °C. Prepared samples were then loaded into the wells and separated in a Mini-PROTEAN 3 cell (Bio-Rad, Hercules, CA, USA). The running buffer consisted of an aqueous solution contained 1.44% *(w/v)* glycine, 0.67% tris-base, and 0.1% SDS. Running conditions were 80 V for the first 20 min and then 150 V until electrophoresis was complete. The PageRuler^TM^ unstained protein ladder was also used as a molecular weight (MW) indicator (Thermo Fisher Scientific, San Jose, CA, USA). After electrophoresis, gel was stained overnight with Coomassie dye PhastGel Blue R-350 (Solon, Ohio, USA). Then, the gel was unstained by using a solution composed of 25% ethanol and 8% acetic acid. Finally, the gel was washed with 50% methanol *(v/v)* and scanned at 200 dpi.

### 4.4. In-Solution Protein Digestion with Trypsin

Protein digestion with trypsin was performed as described by Carrera et al. [25]. Briefly, a total of 100 µg of proteins per sample was dried using a speed vac (Gyrozen). Dried proteins were then denatured in 8 M urea with 25 mM ammonium bicarbonate (pH 8.0) at a protein concentration of 4 µg/µL. Reactions were reduced by adding freshly prepared dithiothreitol (DTT) at a final concentration of 10 mM from 100 mM (10×) stock solution and incubated in a UPV Hybridizer Oven at 56 °C for 45 min with a gentle agitation. Iodoacetamide (IAA) was added up to 50 mM from a 10× stock (500 mM) solution into the reaction tube for alkylation, subsequently incubated with a gentle shaking at room temperature in the dark for 60 min. Then samples were diluted 4-fold using 25 mM ammonium bicarbonate, with a pH of 8.25. Finally, proteins were digested with trypsin (Promega, Madison, WI, USA) overnight at 37 °C in the proportion of 1:100 for the protease enzyme to protein ratio. To stop the tryptic digestion, peptides were acidified with 5% formic acid until pH 2. Samples were preserved at −80 °C until use.

### 4.5. Shotgun LC-MS/MS Analysis

Digested samples were purified for LC-MS/MS analysis following the desalting method [56] by using the C_18_ MicroSpin^TM^ column (The Nest Group, South-borough, MA, USA). Speed vac dried peptides samples were resuspended in 0.1% formic acid, which were analyzed by liquid chromatography-tandem mass spectrometry (LC-MS/MS) using a Proxeon EASY-nLC II liquid chromatography system (Thermo Fisher Scientific, San Jose, CA, USA) coupled with an LTQ-Orbitrap Elite mass spectrometer (Thermo Fisher Scientific).

Peptide separation (1 µg) was performed on a reverse phase (RP) column (EASY-Spray column, 50 cm × 75 µm ID, PepMap C18, 2 µm particles, 100 Å pore size, Thermo Fisher Scientific) with a 10 mm pre-column (Accucore XL C18, Thermo Fisher Scientific) using 0.1% formic acid (mobile phase A) and 98% acetonitrile (98% ACN) with 0.1% formic acid (mobile phase B). A 120 min linear gradient from 5 to 35% B at a flow rate of 300 nL min^−1^ was used. A spray voltage of 1.95 kV and a capillary temperature of 230 °C were used for ionization. The peptides were analyzed in a positive mode (1 µscan; 400–1600 amu), followed by 10 data-dependent higher energy collision dissociation (HCD) MS/MS scans (1 µscans) using a normalized collision energy of 35% and an isolation width of 3 amu. Dynamic exclusion for 30 s after the second fragmentation event was applied, and unassigned charged ions were excluded from the analysis. A total of three biological samples obtained by the above described different extraction methods (OVI1: syringe and purification; OVI2: milking; OVI3: milking and purification) were independently analyzed.

### 4.6. Processing of the Mass Spectrometry Data

All the MS/MS spectra obtained in the LTQ-Orbitrap Elite instrument were analyzed using the search engine SEQUEST-HT (Proteome Discoverer 2.4 package, Thermo Fisher Scientific) against the Cephalopoda UniProtKB database (125,800 protein sequence entries) and the UniGene transcriptome database of *O. vulgaris* paralarvae [3], containing 77,838 protein sequence entries. The following restrictions were used: tryptic cleavage with up to 2 missed cleavage sites and tolerances of 10 ppm for parent ions and 0.06 Da for MS/MS fragment ions. The carbamidomethylation of Cys (C*) was considered a fixed modification. The permissible variable modifications were methionine oxidation (Mox) and acetylation of the N-terminus of the protein (N-Acyl). The results were subjected to statistical analysis to determine the peptide false discovery rate (FDR) using a decoy database and the Target Decoy PSM Validator algorithm [95]. The FDR was kept below 1% for further analysis. Only proteins that matched selected prerequisites were submitted, such as (a) proteins classified as master proteins, (b) proteins with at least 2 unique peptides, and (c) characterized proteins. For the relative protein abundance determination for each sample, a label-free quantification (LFQ) method was used by applying the Minora Feature Detector node and the ANOVA (individual proteins) method included in the Proteome Discoverer 2.4 software (Thermo Fisher Scientific). Peak areas of ion features from the same peptide for different charge forms were accumulated to one value. All the proteins obtained by the 3 different methodologies were used to create a reference dataset of ink proteome.

### 4.7. Functional Gene Ontologies and Pathways Analysis

A final list of all non-redundant protein IDs identified in the group of the 3 ink samples was selected for further bioinformatics analysis. All the homologous genes were identified through UniProt and the NCBI database and were submitted to the PANTHER program version 17.0 (http://www.pantherdb.org/, accessed on 3 June 2022) to classify the proteins based on the 3 main types of annotation: molecular functions, biological processes, and protein classes. Additionally, the pathways involved were also studied using PANTHER classification. According to Thomas et al. [96] and Mi et al. [97], a statistical significance of representation for the analysis was also provided. KEGG pathway analysis was performed by comparing the input data with the background of the *O. bimaculoides* genome by DAVID version 6.8 (https://david.ncifcrf.gov/, accessed on 3 June 2022). Protein functional domains were also identified by InterPro Motifs using the same software comparing the input data with the background of *O. bimaculoides.*

### 4.8. Network Analysis

All the protein networks for octopus ink proteomes were analyzed using STRING (Search Tool for the Retrieval of Interacting Genes) software (v.11.5) (http://stringdb.org/, accessed on 3 June 2022) [98]. This is a large database of known and predicted protein–protein interactions (PPI). Proteins were represented with nodes. The interactions between proteins were represented with continuous lines to represent direct interactions (physical) and with dotted lines to represent indirect interactions (functional). To minimize false positives and false negatives, all interactions tagged as confidence ≥ 0.7 in STRING software were used for the analysis. Cluster networks were created using the MCL (Markov Cluster Algorithm) inflation algorithm, a distance matrix that is included in the STRING website. MCL inflation was set to 1.8 to reduce the number of clusters for all the analyses.

### 4.9. Bioactive Peptides Prediction

Bioactive peptides encrypted in the *O. vulgaris* ink proteins were yielded using different in silico protein hydrolysates with pepsin and trypsin enzymes. For that, all the computational proteolytic digestions were carried out using the MS-Digest software, which is included on the ProteinProspector v. 6.4.0 website (https://prospector.ucsf.edu/prospector/cgi-bin/msform.cgi?form=msdigest, accessed on 9 June 2022). To determine the potential bioactive peptides, all the peptides were ranked using the PeptideRanker software (http://distilldeep.ucd.ie/PeptideRanker/, accessed on 9 June 2022) using the N-to-1 neural network probability. Moreover, all possible peptides were also compared to previous databases integrated in the BIOPEP-UWM portal (https://biochemia.uwm.edu.pl/biopep-uwm/, accessed on 20 June 2022) that included known antimicrobial bioactive peptides such as CAMP_R3_ database (http://www.camp.bicnirrh.res.in/prediction.php, accessed on 3 June 2022) [77]. Additionally, AllerTop v.2 (https://www.ddg-pharmfac.net/AllerTOP/, accessed on 24 June 2022) and ToxinPred (https://webs.iiitd.edu.in/raghava/toxinpred/protein.php, accessed on 24 June 2022) webtools were used to predict the allergenicity and toxicity of peptides [89,90].

## 5. Conclusions

A wide range of proteins were identified from the common octopus (*O. vulgaris*) ink samples for the first time using a shotgun proteomic strategy, indicating that the ink proteome could act as a great reservoir of diverse proteins. A total of 1432 different peptides and 361 non-redundant proteins were identified. Different in silico analyses, including GO word enrichment, pathways, and network investigations, were used to explore the final proteome compilation. Peroxidase, hemocyanin, and CD109 proteins, which are part of the innate immune system, were detected with high percentages of protein coverage and peptide spectrum matches. The most prominent protein classes of octopus ink proteomes were oxidoreductase, transferase, and hydrolase, which seems to indicate that binding and catalytic activities were the main molecular functions, including some crucial immunological activities. The majority of ink proteins were clustered under the metabolic pathways, ribosome and proteasome pathways, xenobiotics and drug metabolic pathways, immune functioning protein networks in the endoplasmic reticulum, and cytoskeletal proteins networks. Ink protein networks contained a variety of immunological proteins associated with the innate immune system, including ferritin, catalase, proteasome, Cu/Zn superoxide dismutase, calreticulin, disulfide isomerase, heat shock protein, etc. Octopus ink proteins release a wide range of potential bioactive peptides after in silico digestion with trypsin and pepsin, which could be used in the future for their antimicrobial, antiviral, and anticancer properties or as potential immune stimulators. The combination of global proteomic findings and the bioinformatics analysis of the octopus ink proteome demonstrates a comprehensive knowledge of this fishery discard and provides potential bioactive peptides of this marine by-product for future study.

## Figures and Tables

**Table 1:** Summary of samples.

Samples	Initial Concentration (µg/µL)	Final Concentration (µg/µL)	Collection Method	Required Protein Purification
OVI1	0.538	0.1698	Syringe	Once
OVI2	0.431	0.431	Milking	No Purification
OVI3	1.381	0.413	Milking	Twice

**Table 2:** Selected potential bioactive peptides of the *O. vulgaris* ink proteome predicted by in silico digestions with trypsin.

Protein	Peptide Sequence	PR Value	Peptide Length	CAMP	AMP Probability	Allergenicity	Toxicity
Protein-glutamine gamma-glutamyltransferase K OS = *Octopus vulgaris*	FGQCWVFSGVLTTGIYCCGPCPVK	0.999637	24	AMP	0.961	Allergen	Toxin
Prominin-1-A isoform X4 OS = *Octopus vulgaris*	IVLYFIGYSICVAIGILFIILIPLIGCCLCCCR	0.999478	33	NAMP	0.132	Non-Allergen	Toxin
PREDICTED: Mucin-3A-like OS = *Aplysia californica*	FISSIAGGIGAAVVLIFLIIIVALCCK	0.999199	27	NAMP	0.401	Non-Allergen	Toxin
Tetraspanin OS = *Octopus vulgaris*	YLMFAFNFIFWLLGCAILGVGIWIR	0.99917	25	NAMP	0.016	Non-Allergen	Non-Toxin
Prosaposin isoform X1 OS = *Octopus vulgaris*	MSFNSLFLVWLGILGCAFGSTTR	0.999078	23	NAMP	0.068	Non-Allergen	Non-Toxin
Neutral protease isoform X1 OS = *Octopus vulgaris*	MHLSVLLYCWYLLFGSLLLIK	0.998754	21	NAMP	0.042	Non-Allergen	Non-Toxin
Tetraspanin OS = *Octopus vulgaris*	SQCLLASFFICLFIIFAILLGAGIFAIISK	0.998242	30	NAMP	0.21	Non-Allergen	Non-Toxin
S-(hydroxymethyl)glutathione dehydrogenase OS = *Octopus vulgaris*	GSNCAVWGLGAVGLAVAMGCK	0.998195	21	AMP	0.911	Non-Allergen	Non-Toxin
Prolyl endopeptidase OS = *Octopus bimaculoides*	TPLDYLNCIVFIFLCHLQPTCR	0.997642	22	NAMP	0.25	Non-Allergen	Non-Toxin
Protein-glucosylgalactosylhydroxylysine glucosidase isoform X2 OS = *Octopus vulgaris*	MLLVVCLLLLTCLTGQVSATSDSYSSTR	0.99758	28	NAMP	0.021	Non-Allergen	Non-Toxin
Neuroglian isoform X1 OS = *Octopus vulgaris*	WIALIVALVLFFIIFILLLCILFNR	0.995036	25	NAMP	0	Non-Allergen	Non-Toxin
Insulin-like growth factor-binding protein complex acid labile subunit isoform X7 OS = *Octopus vulgaris*	LTFALILSMSFCLESNAASDICSTCSCR	0.994916	28	AMP	0.588	Allergen	Non-Toxin
Hemocyanin 1-like OS = *Octopus vulgaris*	IPCLFAIVFAFWLCGYIAEGNLIR	0.994658	24	NAMP	0.212	Non-Allergen	Non-Toxin
Uncharacterized protein isoform X1 OS = *Octopus vulgaris*	MSFGIVLLFVTVVSSLVTAAPLNK	0.993617	24	NAMP	0.274	Allergen	Non-Toxin
Hemocyanin G-type, units Oda to Odg OS = *Octopus vulgaris*	IPCLFAIVFAFWLCGHIAEGNLIR	0.993578	24	AMP	0.59	Non-Allergen	Non-Toxin
Calumenin-like isoform X2 OS = *Aplysia californica*	YYSFFLTIFLFATTLCSTIPKPK	0.993298	23	NAMP	0.06	Non-Allergen	Non-Toxin
Hypothetical protein LOTGIDRAFT_233221 OS = *Lottia gigantea*	QSCIGLILGTGCNVCYIENVK	0.992983	21	AMP	0.699	Allergen	Toxin
Retinal dehydrogenase 2 OS = *Octopus vulgaris*	IMTFTNAIQAGTVWVNTYCCVACQAPFGGFK	0.992039	31	NAMP	0.439	Non-Allergen	Non-Toxin
Transketolase OS = *Octopus vulgaris*	FIECYIAEQNLVGVGIGCACR	0.991698	21	AMP	0.929	Allergen	Toxin
Acid ceramidase-like OS = *Octopus vulgaris*	CPDPCWPW	0.991404	8	NAMP	0.41	Non-Allergen	Toxin
Prominin-1-A isoform X4 OS = *Octopus vulgaris*	TYVTCLVILNTIILFAVVCTFITNELYK	0.990938	28	NAMP	0.179	Non-Allergen	Non-Toxin
Peroxidase-like protein, partial OS = *Euprymna scolopes*	TTMIRPSLILLLAILPCIVLCLTPLQDK	0.990735	28	NAMP	0.018	Non-Allergen	Non-Toxin
Hemocyanin G-type, units Oda to Odg-like OS = *Octopus vulgaris*	SPWLLGATILCIISIFVPVITNGK	0.989103	24	AMP	0.813	Non-Allergen	Non-Toxin
Thyroglobulin isoform X2 OS = *Octopus vulgaris*	YIFFIALSVVAAGAHICSPNACK	0.988906	23	AMP	0.917	Non-Allergen	Non-Toxin
Dipeptidyl peptidase 2 isoform X1 OS = *Octopus vulgaris*	ITGLIWVSLLLILSNGPIGSSADGNNGHNVR	0.988758	31	AMP	0.882	Non-Allergen	Non-Toxin
Hephaestin-like protein OS = *Acropora millepora*	NMSHSLWTSFFLCMLGIVSQVK	0.987807	22	NAMP	0.139	Allergen	Non-Toxin
Uncharacterized protein OS = *Octopus bimaculoides*	LALVLLVLLPLALSASLGESESETAK	0.987554	26	AMP	0.558	Non-Allergen	Non-Toxin
Alpha-mannosidase OS = *Octopus vulgaris*	VFCIFLSFLLVVGNQAYPFHSQSCGYESCNPVK	0.987311	33	NAMP	0.108	Non-Allergen	Non-Toxin
Tetraspanin OS = *Octopus vulgaris*	IAAAGLALAFIQVIGIVFACCLAQAIR	0.984503	27	AMP	0.744	Non-Allergen	Non-Toxin
Peroxidase-like protein (Fragment) OS = *Euprymna scolopes*	LFLVVLPCLVSCLTPITDDLCQK	0.984059	23	AMP	0.519	Non-Allergen	Non-Toxin
Xaa-Pro dipeptidase-like OS = *Aplysia californica*	LNDGDACLLDMGTEYCCYASDITCSYPVNGK	0.982857	31	NAMP	0.144	Allergen	Toxin
AIFM3 OS = *Sepia pharaonis*	SVPFFWSMMFGK	0.980626	12	NAMP	0.017	Allergen	Non-Toxin
Short-chain collagen C4-like OS = *Octopus vulgaris*	DDGGAVLYFVQSVCGSLPCPPYVK	0.978133	24	NAMP	0.429	Allergen	Non-Toxin
Hemocyanin G-type (Fragment) OS = *Enteroctopus dofleini*	ILCLFAFVFAFWLSGQSAEGNLIR	0.977782	24	AMP	0.551	Non-Allergen	Non-Toxin
Thyroglobulin isoform X2 OS = *Octopus vulgaris*	CETDGTFSAVQCHGSVCYCAHTDGTR	0.976203	26	NAMP	0.149	Allergen	Toxin
Mucin-5AC-like isoform X1 OS = *Octopus vulgaris*	SSFDGGSFGGGIAAGIAIAILLLALIYLFYR	0.976129	31	AMP	0.88	Non-Allergen	Non-Toxin
26S proteasome non-ATPase regulatory subunit 2 OS = *Octopus vulgaris*	SLMSPVAVAGLLSVLISCLDVK	0.975381	22	NAMP	0.054	Allergen	Non-Toxin
Mucin-3A-like OS = *Aplysia californica*	FEFPFR	0.973591	6	NAMP	0.02	Non-Allergen	Non-Toxin
MYH OS = *Sepia pharaonis*	NWQWWR	0.973264	6	AMP	0.959	Non-Allergen	Non-Toxin
DNAH OS = *Sepia pharaonis*	CYLCLMGALQLDLGGAPAGPAGTGK	0.971438	25	AMP	0.693	Non-Allergen	Non-Toxin
Tetraspanin OS = *Octopus vulgaris*	EHNVCTMVFAVLLALIFILQLAGGIAAFVMR	0.969205	31	NAMP	0.082	Non-Allergen	Non-Toxin
Microtubule-associated protein futsch isoform X4 OS = *Octopus vulgaris*	ACFWDFTR	0.968121	8	NAMP	0.003	Allergen	Non-Toxin
Apoptosis-inducing factor 3 isoform X1 OS = *Octopus vulgaris*	SVPCFWTMMFGK	0.967302	12	NAMP	0.01	Non-Allergen	Non-Toxin
Probable methylmalonate-semialdehyde dehydrogenase [acylating], mitochondrial OS = *Octopus vulgaris*	GLQVVETCCSLSATCLGETLTGIAK	0.967224	25	AMP	0.937	Non-Allergen	Toxin
H(+)-transporting two-sector ATPase OS = *Octopus vulgaris*	FCPFYK	0.966449	6	NAMP	0.328	Allergen	Non-Toxin
Dehydrogenase/reductase SDR family member 4 isoform X1 OS = *Octopus vulgaris*	APFLFCK	0.965743	7	AMP	0.642	Allergen	Non-Toxin
Carboxypeptidase OS = *Octopus vulgaris*	LYANLLSSCCGSNTTVCYISK	0.965007	21	AMP	0.617	Non-Allergen	Toxin
Hemocyanin subunit 1 OS = *Euprymna scolopes*	VFVGFLLHGFGSSAYATFDICNDAGECR	0.96087	28	NAMP	0.087	Non-Allergen	Non-Toxin
Ferritin OS = *Octopus vulgaris*	GFFEFFK	0.959334	7	NAMP	0.065	Non-Allergen	Non-Toxin
Dystrophin isoform X4 OS = *Octopus vulgaris*	CIIMYIMCLFQVLQNSSNNSSNETNTK	0.95929	27	NAMP	0.052	Non-Allergen	Toxin
Glutathione S-transferase A-like OS = *Crassostrea gigas*	SWPPHWK	0.957413	7	AMP	0.691	Allergen	Non-Toxin
Xylose isomerase-like OS = *Crassostrea gigas*	FSVCFWHTFR	0.955452	10	NAMP	0.012	Non-Allergen	Non-Toxin
Malate dehydrogenase OS = *Octopus vulgaris*	DDLFNTNASIVGNLADACAQFCPK	0.953131	24	NAMP	0.461	Non-Allergen	Non-Toxin
Uncharacterized protein OS = *Octopus vulgaris*	MGWYMR	0.952753	6	NAMP	0.002	Non-Allergen	Non-Toxin
H(+)-transporting two-sector ATPase OS = *Octopus vulgaris*	VLDALFPCVQGGTTAIPGAFGCGK	0.952067	24	AMP	0.958	Non-Allergen	Non-Toxin
Myosin heavy chain, striated muscle OS = *Octopus vulgaris*	NWEWWR	0.951523	6	NAMP	0.478	Non-Allergen	Non-Toxin
Rab GDP dissociation inhibitor OS = *Sepia pharaonis*	DDFSFFFFSFSFPR	0.94971	14	NAMP	0.065	Allergen	Non-Toxin
Uncharacterized protein OS = *Octopus vulgaris*	FSEQEWLFFCMK	0.949352	12	NAMP	0.004	Allergen	Non-Toxin
Zinc finger ZZ-type and EF-hand domain-containing protein 1 OS = *Octopus vulgaris*	LFPSLPFR	0.949115	8	NAMP	0.258	Non-Allergen	Non-Toxin
Uncharacterized protein OS = *Octopus vulgaris*	DWFYMTGFK	0.949111	9	NAMP	0.001	Allergen	Non-Toxin
Filamin-A isoform X1 OS = *Octopus vulgaris*	AIGALVDACGPGLCPDWADWAPK	0.948884	23	AMP	0.869	Non-Allergen	Non-Toxin
Uncharacterized protein (Fragment) OS = *Octopus bimaculoides*	YASNFLWPFK	0.947804	10	NAMP	0.019	Allergen	Non-Toxin
Glyoxalase I OS = *Octopus vulgaris*	FDFPPLK	0.946351	7	NAMP	0.029	Allergen	Non-Toxin
Hypothetical protein CAPTEDRAFT_117881, partial OS = *Capitella teleta*	NENGALLYFVQAVCGSLPCPPYVNGR	0.945612	26	AMP	0.509	Non-Allergen	Non-Toxin
Thioredoxin isoform X2 OS = *Octopus vulgaris*	LIIIDFFATWCGPCK	0.944114	15	NAMP	0.467	Allergen	Non-Toxin
Glyoxylate reductase/hydroxypyruvate reductase OS = *Octopus vulgaris*	NGNWGLWKPMWILGSSFANR	0.942087	20	AMP	0.645	Non-Allergen	Non-Toxin
Zinc finger ZZ-type and EF-hand domain-containing protein 1 OS = *Octopus vulgaris*	CLQCSALDFCASCITGGCFK	0.941639	20	AMP	0.997	Allergen	Toxin
Prominin-1-A isoform X4 OS = *Octopus vulgaris*	SVAVPCSVLLLWILIAFSLVDHSFAQNSSQQHR	0.941512	33	NAMP	0.081	Non-Allergen	Non-Toxin
Chorion peroxidase-like OS = *Octopus vulgaris*	QWCGLSFPR	0.940111	9	NAMP	0.018	Allergen	Non-Toxin
Hypothetical protein ACA1_115170 OS = *Acanthamoeba castellanii* str. Neff	CHFVFLALAPFMPK	0.939609	14	NAMP	0.134	Allergen	Non-Toxin
MYH OS = *Sepia pharaonis*	YYSGLIYTYSGLFCVVVNPYK	0.939159	21	NAMP	0.061	Allergen	Non-Toxin
Hypothetical protein BRAFLDRAFT_127655 OS = *Branchiostoma floridae*	FAIVLCLASVAYGCCAPEYFTAHTLIR	0.939147	27	AMP	0.615	Non-Allergen	Toxin
1,2-dihydroxy-3-keto-5-methylthiopentene dioxygenase OS = *Octopus vulgaris*	FPNFDNMMK	0.934874	9	NAMP	0.099	Non-Allergen	Non-Toxin
Polyol dehydrogenase OS = *Octopus vulgaris*	AGVGINSTVLISGAGPIGLCCFLTAK	0.934366	26	AMP	0.995	Allergen	Toxin
Spectrin alpha chain isoform X2 OS = *Octopus vulgaris*	EFSMMFR	0.931708	7	NAMP	0.001	Non-Allergen	Non-Toxin
Puromycin-sensitive aminopeptidase-like OS = *Crassostrea gigas*	DHWQFFCER	0.93076	9	NAMP	0	Allergen	Non-Toxin
Tetratricopeptide repeat protein 38 OS = *Octopus bimaculoides*	DWSVCGMLACHNYWHWALYHIEK	0.930471	23	NAMP	0.04	Allergen	Non-Toxin
Glucose-6-phosphate 1-dehydrogenase OS = *Octopus vulgaris*	IYPTLWCLFR	0.930391	10	NAMP	0.052	Allergen	Non-Toxin
Cilia- and flagella-associated protein 65 OS = *Sepia pharaonis*	IDLFHLFCL	0.927866	9	NAMP	0.458	Non-Allergen	Non-Toxin
Hemocyanin subunit 1 OS = *Euprymna scolopes*	LNHLPLLCLAVILTLWMSGSNTVNGNLVR	0.926117	29	NAMP	0.316	Non-Allergen	Non-Toxin
Uncharacterized protein OS = *Octopus vulgaris*	LWFDKPPHFR	0.925721	10	NAMP	0.009	Allergen	Non-Toxin
Trans-1,2-dihydrobenzene-1,2-diol dehydrogenase isoform X1 OS = *Octopus vulgaris*	SGLIDIGVYLIWLANFIFK	0.925485	19	NAMP	0.493	Non-Allergen	Non-Toxin
Thyroglobulin isoform X2 OS = *Octopus vulgaris*	GFCGCCDICIK	0.925073	11	AMP	0.901	Non-Allergen	Toxin
Protein-glucosylgalactosylhydroxylysine glucosidase isoform X2 OS = *Octopus vulgaris*	QADVILLGFPLMMNMPK	0.922173	17	NAMP	0.03	Non-Allergen	Non-Toxin
Prostaglandin reductase 1-like OS = *Crassostrea gigas*	SGETVLVNAAAGAVGSIVGQIAK	0.922086	23	AMP	0.962	Non-Allergen	Non-Toxin
Pacifastin domain-containing protein OS = *Octopus bimaculoides*	DDCNLCFCGANGAVSCTK	0.921918	18	AMP	0.984	Allergen	Non-Toxin
TGc domain-containing protein OS = *Octopus bimaculoides*	ESFILLFNPWCK	0.9189	12	NAMP	0.029	Non-Allergen	Non-Toxin
Dystrophin isoform X4 OS = *Octopus vulgaris*	CFNFDVCQNCFFSGR	0.918754	15	AMP	0.614	Allergen	Non-Toxin
Inter-alpha-trypsin inhibitor heavy chain H3-like OS = *Crassostrea gigas*	LLFVMLGAVFYLGMTANGEPR	0.918111	21	NAMP	0.026	Non-Allergen	Non-Toxin
Xylose isomerase-like OS = *Crassostrea gigas*	FCCLYIFNK	0.916888	9	AMP	0.761	Non-Allergen	Toxin
UPF0462 protein C4orf33 homolog OS = *Saccoglossus kowalevskii*	GQFDFPDFHR	0.916554	10	NAMP	0.015	Allergen	Non-Toxin
Xylose isomerase-like OS = *Crassostrea gigas*	LGAENFVFWGGR	0.916397	12	NAMP	0.01	Allergen	Non-Toxin
PREDICTED: Puromycin-sensitive aminopeptidase-like OS = *Crassostrea gigas*	AFPCWDEPSFK	0.91609	11	NAMP	0.003	Non-Allergen	Non-Toxin
Uncharacterized protein isoform X1 OS = *Octopus vulgaris*	FSGPWYTIWK	0.915775	10	NAMP	0.341	Allergen	Non-Toxin
Leukotriene A-4 hydrolase-like OS = *Octopus vulgaris*	MEFFFK	0.915244	6	NAMP	0.065	Non-Allergen	Non-Toxin
Zinc finger ZZ-type and EF-hand domain-containing protein 1 OS = *Octopus vulgaris*	MLPPQPLFNPMK	0.915243	12	NAMP	0.157	Non-Allergen	Non-Toxin
S-formylglutathione hydrolase OS = *Octopus bimaculoides*	SVSAFAPICNPVNCNWGK	0.915182	18	AMP	0.775	Non-Allergen	Non-Toxin
DNAH OS = *Sepia pharaonis*	IPVFANFWK	0.913394	9	NAMP	0.277	Allergen	Non-Toxin
Aconitate hydratase, mitochondrial OS = *Octopus vulgaris*	LTETLEAIDGCVLANACGPCIGQWDR	0.910547	26	AMP	0.583	Non-Allergen	Non-Toxin
Inter-alpha-trypsin inhibitor heavy chain H3-like OS = *Crassostrea gigas*	DTLPNIPGIFIKPFSCSNNLCLR	0.910355	23	AMP	0.782	Non-Allergen	Non-Toxin
Hemocyanin subunit 2 (Fragment) OS = *Sepia officinalis*	VFGGFWLHGIK	0.907156	11	AMP	0.739	Non-Allergen	Non-Toxin
Uncharacterized protein OS = *Octopus bimaculoides*	SSLCFLQWTHFR	0.907105	12	NAMP	0.002	Non-Allergen	Non-Toxin
Glutathione S-transferase omega OS = *Octopus vulgaris*	FLSAWYCPFAQR	0.906935	12	NAMP	0.015	Allergen	Non-Toxin
GDP-4-keto-6-deoxy-D-mannose-3,5-epimerase-4-reductase OS = *Octopus vulgaris*	YNLDFFR	0.906435	7	NAMP	0.002	Non-Allergen	Non-Toxin
Hemocyanin subunit 3 OS = *Sepia officinalis*	TSFLFLAFVATSWFVYAVTASK	0.905214	22	NAMP	0.414	Non-Allergen	Non-Toxin
Peroxidase-like protein (Fragment) OS = *Euprymna scolopes*	TCLTPATGACSCDGVPAETQIGQCNVFGPAA	0.904898	31	AMP	0.617	Allergen	Non-Toxin
Cathepsin L1 OS = *Octopus vulgaris*	NSWGGSWGMK	0.904726	10	NAMP	0.095	Non-Allergen	Non-Toxin
Hemocyanin subunit 1 OS = *Euprymna scolopes*	VFAGFLFMGIK	0.904542	11	AMP	0.865	Non-Allergen	Non-Toxin
Hemocyanin G-type, units Oda to Odg-like OS = *Octopus vulgaris*	MFAGFLLK	0.902865	8	AMP	0.512	Non-Allergen	Non-Toxin
Hemocyanin G-type, units Oda to Odg OS = *Octopus vulgaris*	YACCLHGMPVFPHWHR	0.90265	16	NAMP	0.012	Non-Allergen	Toxin
Zinc finger ZZ-type and EF-hand domain-containing protein 1 OS = *Octopus vulgaris*	MINFLLHQGACNVEYGNTQQACTIACMIQR	0.900661	30	NAMP	0.052	Non-Allergen	Non-Toxin

**Table 3:** Selected potential bioactive peptides of the *O. vulgaris* ink proteome predicted by in silico digestion with pepsin.

Protein	Sequence	PR Value	Peptide Length	CAMP	AMP Probability	Allergenicity	Toxicity
Prominin-1-A isoform X4 OS = *Octopus vulgaris*	CCCRCCNRCGGRHMKY	0.970522	16	AMP	0.988	Allergen	Toxin
Adenylyl cyclase-associated protein 1 isoform X4 OS = *Octopus vulgaris*	PPPPPPPPPPPPA	0.966356	13	AMP	0.898	Non-Allergen	Non-Toxin
Uncharacterized protein isoform X1 OS = *Octopus vulgaris*	PPPPPPPPPSKPNHPPPVGL	0.96064	20	AMP	0.768	Non-Allergen	Toxin
Retinal dehydrogenase 2 OS = *Octopus vulgaris*	CMGQCCF	0.95616	7	AMP	0.858	Allergen	Toxin
Ganglioside GM2 activator OS = *Octopus vulgaris*	PQCPQPF	0.947135	7	AMP	0.517	Allergen	Non-Toxin
Acid ceramidase-like OS = *Octopus vulgaris*	QKCPDPCW	0.944659	8	NAMP	0.01	Non-Allergen	Toxin
Hemocyanin subunit 2 (Fragment) OS = *Sepia officinalis*	SDPMRPF	0.938433	7	AMP	0.879	Allergen	Non-Toxin
Hypothetical protein ACA1_115170 OS = *Acanthamoeba castellanii* str. Neff	CGVCPKCHF	0.933815	9	AMP	0.978	Non-Allergen	Non-Toxin
Hemocyanin subunit 2 (Fragment) OS = *Sepia officinalis*	KKPMMPF	0.932566	8	AMP	0.978	Allergen	Non-Toxin
NADH-cytochrome b5 reductase OS = *Octopus vulgaris*	MCGPPPMI	0.930352	8	NAMP	0.002	Allergen	Toxin
Heat shock cognate 71 kDa protein OS = *Octopus vulgaris*	GGMPGGMPGGMPGGMPNF	0.92432	18	AMP	0.504	Allergen	Non-Toxin
Ras GTPase-activating protein-binding protein 2 OS = *Octopus vulgaris*	GQPMRRF	0.920825	7	NAMP	0.486	Non-Allergen	Non-Toxin
AAA domain-containing protein OS = *Octopus bimaculoides*	GPPGCGKTML	0.909643	10	NAMP	0.192	Non-Allergen	Non-Toxin
Ecdysteroid-regulated 16 kDa protein OS = *Danaus plexippus*	CRNDCGCVSCVCL	0.907811	13	AMP	0.813	Allergen	Toxin
N-acyl-L-amino-acid amidohydrolase OS = *Octopus bimaculoides*	KCPGNPGHGSRF	0.901021	12	NAMP	0.135	Allergen	Non-Toxin

## Data Availability

Data available in publicly accessible repositories. The data presented in this study are openly available in [MassIVE, (https://massive.ucsd.edu/, accessed on 3 June 2022)], reference number [MSV000089896] and [ProteomeXchange database, (https://www.proteomexchange.org/, accessed on 3 June 2022)], reference number [PXD035359]. The original data presented in the study are also included in the article/Supplementary Material; further inquiries can be directed to the corresponding authors.

## Supplementary Materials

The following supporting information can be downloaded at: https://www.mdpi.com/article/10.3390/md21040206/s1, File S1: Tables of Peptide Spectrum Matches (PSMs), Peptide Groups and Proteins; File S2: Common Octopus (*O. vulgaris*) Ink Proteome (FDR < 1%); File S3: Gene Level 1 and Level 2 PANTHER Analysis of Octopus Ink Proteome (Protein Class, Biological Process, Molecular Functions) and Tables of DAVID Analysis; File S4: KEGG pathway analysis of octopus ink proteome by DAVID version 6.8; File S5: InterPro Motifs analysis of the *O. vulgaris* ink proteome by DAVID version 6.8; File S6: MCL Clustered Proteins Descriptions (STRING v.11.5); File S7: Potential Bioactive Peptides Predicted After Pepsin and Trypsin Digestion. Supplementary Figures S1 and S2: Molecular Function and Biological Process, respectively of the octopus ink proteome identified by shotgun proteomics and categorized by PANTHER (http://pantherdb.org/, accessed on 3 June 2022) using the homologous gene list.
