# Supplementary material for: Insights into Common Octopus (Octopus vulgaris) Ink Proteome and Bioactive Peptides Using Proteomic Approaches
Source: Mar Drugs. 2023 Mar 24;21(4):206. doi: 10.3390/md21040206 (PMC10142993; doi:10.3390/md21040206)
Supplement: Supplementary file 1 [file marinedrugs-21-00206-s001.zip › Supplementary File S2.pdf]

Supplementary File S2: Common octopus (*O. vulgaris*) ink proteome (FDR < 1%)

Nº	*Accession*	*Description*	*Gene name*	*Homologous Gene*	*PSMs*	*UPs*	*Cov. [%]*
1.	A0A6P7SER3	Hemocyanin 1-like OS=*Octopus vulgaris*	LOC115212215	HCA	527	16	47
2.	A0A6P7SDC4	Hemocyanin G-type, units Oda to Odg OS=*Octopus vulgaris*	LOC115211670	HCG	517	2	51
3.	Cluster-58773.162319	Hemocyanin G-type OS=*Enteroctopus dofleini*	ODHCY	HCG	512	4	50
4.	Cluster-58773.132244	Peroxidase-like protein, partial OS=*Euprymna scolopes*	GenBank: L01771.1	prx1	268	15	35
5.	A0A6P7TQ10	Uncharacterized protein OS=*Octopus vulgaris*	LOC115225177		126	25	43
6.	A0A6P7SI10	CD109 antigen isoform X2 OS=*Octopus vulgaris*	LOC115213260	CD109	107	36	37
7.	Q9BJ58	Hemocyanin G-type (Fragment) OS=*Enteroctopus dofleini*	GenBank: AF338426.3	HCG	103	1	13
8.	O61363	Hemocyanin G-type, units Oda to Odg OS=*Enteroctopus dofleini*	ODHCY	HCG	95	1	12
9.	Cluster-58773.88130	PREDICTED: Puromycin-sensitive aminopeptidase-like OS=*Crassostrea gigas*	LOC117689997	NPEPPSL1	58	30	37
10.	A0A6P7U7W9	Putative aminopeptidase W07G4.4 isoform X1 OS=*Octopus vulgaris*	LOC115232621	YH24	36	23	61
11.	A0A6P7T042	LOW QUALITY PROTEIN: Paramyosin-like OS=*Octopus vulgaris*	LOC115218392	PRM	33	4	28
12.	A0A7E6ET70	Tropomyosin Tod p 1.0102 isoform X27 OS=*Octopus vulgaris*	LOC115210626	ov-tm	30	4	49
13.	A0A7E6EW98	10-formyltetrahydrofolate dehydrogenase OS=*Octopus vulgaris*	LOC115216749	ALDH1L2	47	26	34
14.	Cluster-58773.164376	PREDICTED: Paramyosin-like isoform X1 OS=*Crassostrea gigas*	LOC117692239	PRM	33	1	35
15.	A0A7E6EQL5	Tropomyosin Tod p 1.0102 isoform X3 OS=*Octopus vulgaris*	LOC115210626	ov-tm	29	3	49
16.	Cluster-58773.13946	Hypothetical protein BRAFLDRAFT_209115 OS=*Branchiostoma floridae*	N/A	BRAFLDRAFT_209115	40	18	44
17.	Cluster-58773.74875	Peroxidase-like protein, partial OS=*Euprymna scolopes*	LOC115221592	prx1	53	3	30
18.	A0A0L8FXZ4	Uncharacterized protein OS=*Octopus bimaculoides*	OCBIM_22004572mg	OCBIM_22004572mg	117	2	23
19.	A0A7E6EQM0	Tropomyosin Tod p 1.0102 isoform X10 OS=*Octopus vulgaris*	LOC115210626	ov-tm	26	0	48
20.	A0A6P7TRC4	Myosin heavy chain, striated muscle OS=*Octopus vulgaris*	LOC115225111	MHC	28	23	12
21.	A0A0L8G7X1	Uncharacterized protein OS=*Octopus bimaculoides*	OCBIM_22038393mg	OCBIM_22038393mg	31	2	25
22.	A0A0L8FFZ3	Tyrosinase_Cu-bd domain-containing protein (Fragment) OS=*Octopus bimaculoides*	OCBIM_22022789mg	DCT	41	1	30
23.	A0A7E6FFI6	Chorion peroxidase-like OS=*Octopus vulgaris*	LOC115221248	pxt	48	1	30
24.	I0JGT9	Actin I OS=*Sepia officinalis*	ACTI	ACTI	23	2	40
25.	Cluster-58773.163468	Thioester-containing protein 1 OS=*Euprymna scolopes*	GenBank: JQ392569.1	TEP	25	3	34
26.	A0A6P7SKQ4	Actin, cytoplasmic OS=*Octopus vulgaris*	LOC115214120	ACTB	25	2	38
27.	A0A7E6FE51	Filamin-A isoform X1 OS=*Octopus vulgaris* OX=6645	LOC115220251	filip1	18	17	9
28.	A0A0L8H9P1	Uncharacterized protein OS=*Octopus bimaculoides*	OCBIM_22019540mg	OCBIM_22019540mg	19	6	31
29.	A0A6P7TXP9	Uncharacterized protein OS=*Octopus vulgaris*	LOC115231284		17	5	57
30.	Cluster-58773.141566	Fructose-bisphosphate aldolase OS=*Crassostrea gigas*	LOC105335896	fbaA	20	10	23
31.	A0A6P7SAW7	Transketolase OS=*Octopus vulgaris*	LOC115210747	TKT	17	10	19

32.	A0A6P7SD30	Tropomyosin Tod p 1.0102 OS=*Octopus vulgaris*	LOC115211774	ov-tm	13	9	38
33.	Q27Q56	Hemocyanin subunit 2 (Fragment) *OS=Sepia officinalis*	GenBank: DQ388570.1	HCB	25	0	3
34.	A0A7E6F2W5	Calreticulin OS=*Octopus vulgaris*	LOC115215618	CALR	14	9	33
35.	A0A6P7TGG8	Gamma-cystathionase OS=*Octopus vulgaris*	LOC115223921	CTH	18	9	28
36.	A0A812CKE2	ACTB_G1 OS=*Sepia pharaonis*	SPHA_36281	SPHA_36281	15	2	31
37.	A0A6P7T7W6	Aspartyl aminopeptidase OS=*Octopus vulgaris*	LOC115220844	DNPEP	12	9	33
38.	Cluster-58773.104902	PREDICTED: Xaa-Pro dipeptidase-like OS=*Aplysia californica*	N/A	pepQ	17	12	31
39.	A0A0S1U346	Triosephosphate isomerase OS=*Amphioctopus fangsiao*	N/A	TPI1	13	10	43
40.	A0A6P7SDN6	Gelsolin-like protein 2 OS=*Octopus vulgaris*	LOC115211664	GSN	10	9	33
41.	A0A812CBN1	Uncharacterized protein OS=*Sepia pharaonis*	SPHA_33848	SPHA_33848	26	1	3
42.	A0A6P7SJH5	Cytosolic non-specific dipeptidase OS=*Octopus vulgaris*	LOC115213824	CNDP2	17	10	35
43.	Cluster-58773.126821	Fatty acid-binding protein, liver OS=*Acanthisitta chloris*	N310_11520	N310_11520	15	6	34
44.	Cluster-58773.194608	Sodium-calcium exchanger OS=*Doryteuthis pealeii*	GenBank: ACL80558.1	SLC8A1	15	9	45
45.	A0A6P7TGA0	Isocitrate dehydrogenase [NADP] OS=*Octopus vulgaris*	LOC115223822	IDH1	10	8	18
46.	Cluster-58773.122645	Glutathione-S-transferase *OS=Sepiella maindroni*	GST	GST	16	9	40
47.	A0A077B6R8	Hemocyanin subunit 1 OS=*Euprymna scolopes*	HCY1	HCY1	22	1	3
48.	A0A6P7T8U5	Uncharacterized protein OS=*Octopus vulgaris*	LOC115221216		11	3	24
49.	A0A6P7T066	Glyceraldehyde-3-phosphate dehydrogenase OS=*Octopus vulgaris*	LOC115217845	GAPDHS	14	1	18
50.	Cluster-58773.160890	Glyceraldehyde-3-phosphate dehydrogenase OS=*Cancer borealis*	LOC115216358	GAPDHS	13	2	20
51.	A0A2I7NB22	Superoxide dismutase [Cu-Zn] OS=*Octopus vulgaris*	LOC115227776	SOD1	6	3	36
52.	W6CNR9	Hemocyanin subunit 3 OS=*Sepia officinalis*	N/A	HCC	16	1	3
53.	Cluster-58773.135700	Serine protease 1 OS=*Heterololigo bleekeri*	GenBank: AB512499.1		12	3	42
54.	Cluster-58773.162111	Laminin subunit alpha-2, partial OS=*Stegodyphus mimosarum*	X975_11537	lama2	9	2	22
55.	A0A6P7TYN8	Protein disulfide-isomerase OS=*Octopus vulgaris*	LOC115231769	PDI	9	8	21
56.	A0A812CD65	Uncharacterized protein OS=*Sepia pharaonis*	SPHA_33819	SPHA_33819	21	1	2
57.	A0A6P7S9Z4	Calmodulin OS=*Octopus vulgaris*	LOC115210643	CALM	13	5	48
58.	A0A6P7SIX4	Inorganic diphosphatase OS=*Octopus vulgaris*	LOC115213471	NDKB	12	7	24
59.	Cluster-58773.127031	Nucleoside diphosphate kinase B OS=*Crassostrea gigas*	N/A	NME2	11	5	35
60.	A0A6P7SRB2	Acidic mammalian chitinase isoform X1 OS=*Octopus vulgaris*	LOC115215700	CHIA	9	9	26
61.	A0A6P7TMW4	Heat shock protein HSP 90-alpha isoform X2 OS=*Octopus vulgaris*	LOC115224220	HSP90AA1	10	10	14
62.	Cluster-58773.162759	Uncharacterized protein LOC101847027 isoform X1 OS=*Aplysia californica*	LOC101847027		12	8	37
63.	A0A6P7T5D5	Aspartate aminotransferase OS=*Octopus vulgaris*	LOC115219838	GOT1	9	8	20
64.	A0A7E6F5B1	Collagen alpha-1(XII) chain-like OS=*Octopus vulgaris*	LOC118765117	COL12A1	8	7	19
65.	A0A0L8GJH5	Pyruvate kinase OS=*Octopus bimaculoides*	OCBIM_22032473mg	PyK	8	7	13

66.	A0A7E6EUU7	Uncharacterized protein OS=*Octopus vulgaris*	LOC118763595		6	5	54
67.	A0A6P7SUK9	Adenosylhomocysteinase OS=*Octopus vulgaris*	LOC115216611	AHCY	9	8	23
68.	G0Y7C4	Arginine kinase OS=*Sepiella maindroni*	AK	AK	6	1	12
69.	A0A6P7SQQ6	Cathepsin L1 OS=*Octopus vulgaris*	LOC115215566	CTSL1	8	5	11
70.	A0A6P7TKE4	Probable aminopeptidase NPEPL1 isoform X1 OS=*Octopus vulgaris*	LOC115223934	NPEPL1	11	7	21
71.	Cluster-58773.174510	Hypothetical protein BRAFLDRAFT_127655 OS=*Branchiostoma floridae*	N/A	BRAFLDRAFT_127655	9	4	22
72.	Cluster-58773.83035	PREDICTED: Thymidine phosphorylase-like isoform X1 OS=*Haplochromis burtoni*	LOC121814411	TYMP	11	9	18
73.	Cluster-58773.161841	Arginine kinase OS=*Amphioctopus fangsiao*	AK	AK	8	2	44
74.	Cluster-58773.89511	PREDICTED: Alpha-aminoadipic semialdehyde dehydrogenase-like OS=*Aplysia californica*	N/A	ALDH7A1	7	4	15
75.	A0A6P7SYF3	Retinal dehydrogenase 1 OS=*Octopus vulgaris*	LOC115217862	aldh1a2	7	7	17
76.	Cluster-58773.161692	14-3-3 protein epsilon OS=*Octopus bimaculoides*	LOC115213049	YWHAE	7	1	18
77.	A0A6P7TYT0	Histidine--tRNA ligase OS=*Octopus vulgaris*	LOC115230998	HARS1	5	1	11
78.	Cluster-58773.47482	Hypothetical protein CAPTEDRAFT_211623 OS=*Capitella teleta*	GenBank: KB301731.1	CAPTEDRAFT_211623	10	5	27
79.	A0A6P7UAQ3	14 kDa Fatty acid-binding protein OS=*Octopus vulgaris*	LOC115232340	Fabp	11	5	55
80.	Q9TWW9	ALPHAM, Alpha-macroglobulin proteinase inhibitor (Fragment) OS=*Octopus vulgaris*	N/A		6	1	70
81.	A0A6P7SQT5	Cathepsin L1-like OS=*Octopus vulgaris*	LOC115215560	CTSL1	6	4	15
82.	A0A6P7TRF4	Catalase OS=*Octopus vulgaris*	LOC115228352	CAT	7	5	14
83.	A0A7E6FT44	4-hydroxyphenylpyruvate dioxygenase OS=*Octopus vulgaris*	LOC115226087	HPD	6	4	15
84.	A0A7E6EY74	Twitchin OS=*Octopus vulgaris*	LOC115213975	Twitchin	5	5	2
85.	A0A0L8HJ80	Major vault protein OS=*Octopus bimaculoides*	OCBIM_22013292mg	MVP	8	7	8
86.	A0A812DI02	Tubulin beta chain OS=*Sepia pharaonis*	SPHA_52176	TUBB	6	6	22
87.	Cluster-58773.147322	Hypothetical protein LOTGIDRAFT_161773 OS=*Lottia gigantea*	LOTGIDRAFT_161773	LOTGIDRAFT_161773	6	6	12
88.	A0A0L8FRZ6	14_3_3 domain-containing protein OS=*Octopus bimaculoides*	OCBIM_22009637mg	OCBIM_22009637mg	8	1	16
89.	Q06331	Tubulin alpha chain OS=*Enteroctopus dofleini*	LOC115226892	TUBA	5	2	14
90.	A0A6P7SVM2	Spectrin beta chain OS=*Octopus vulgaris*	LOC115216977	SPTBN1	4	4	3
91.	A0A7E6EZ79	L-threonine ammonia-lyase-like OS=*Octopus vulgaris*	LOC115214389	tdcB	5	5	14
92.	A0A812CBL4	HSPA1s OS=*Sepia pharaonis*	SPHA_33061	HSPA1s	6	2	9
93.	Cluster-58773.152500	PREDICTED: Adenosine deaminase-like OS=*Crassostrea gigas*	CGI_10018537	Adal	5	4	16
94.	Cluster-58773.174459	Hypothetical protein LOTGIDRAFT_153262 OS=*Lottia gigantea*	LOTGIDRAFT_153262	LOTGIDRAFT_153262	5	1	13
95.	A0A6P7SP92	Carbonyl reductase (NADPH) OS=*Octopus vulgaris*	LOC115215080	CBR	6	6	25
96.	A0A6P7TG88	Chymotrypsin B-like OS=*Octopus vulgaris*	LOC115222971	Ctrb	7	1	32
97.	A0A6P7TPW3	2-phospho-D-glycerate hydro-lyase OS=*Octopus vulgaris*	LOC115224300	ENO	6	6	12
98.	A0A6P7TH79	Ferritin OS=*Octopus vulgaris*	LOC115223217	FER	5	5	33
99.	Cluster-58773.173059	Calreticulin OS=*Hyriopsis cumingii*	LOC115215618	CALR	3	1	22

100.	Cluster-58773.93343	PREDICTED: Xylose isomerase-like OS=*Crassostrea gigas*	LOC105340946	XYLA	5	5	13
101.	A0A0L8G196	GDP-D-mannose dehydratase (Fragment) OS=*Octopus bimaculoides*	OCBIM_22002213mg	Gmd	6	4	17
102.	Cluster-58773.168901	Aspartate aminotransferase, mitochondrial-like, partial OS=*Crassostrea gigas*	CGI_10027059	GOT2	6	6	19
103.	A0A6P7TJB0	O-methyltransferase MdmC OS=*Octopus vulgaris*	LOC115222932	MdmC	6	6	22
104.	A0A7E6FRZ6	Hemocyanin G-type, units Oda to Odg-like OS=*Octopus vulgaris*	LOC115225590	ODHCY	5	1	2
105.	A0A6P7TWP3	Chymotrypsin-like protease CTRL-1 OS=*Octopus vulgaris*	LOC115226866	CTRL	7	2	14
106.	A0A6P7T7N8	Dihydropteridine reductase OS=*Octopus vulgaris*	LOC115219744	QDPR	4	3	15
107.	Cluster-58773.148525	Hypothetical protein LOTGIDRAFT_201834 OS=*Lottia gigantea*	LOTGIDRAFT_201834	LOTGIDRAFT_201834	7	7	8
108.	A0A6P7T9I0	Glyoxalase I OS=*Octopus vulgaris*	LOC115221453	GLO1	5	3	21
109.	A0A6P7SJV4	S-(hydroxymethyl)glutathione dehydrogenase OS=*Octopus vulgaris*	LOC115213890	ADH5	6	3	9
110.	F0V3W2	Hemocyanin (Fragment) OS=*Lepidochitona cinerea*	hc-fg	hc-fg	11	2	3
111.	R9WBT1	14-3-3 protein epsilon OS=*Octopus bimaculoides*	LOC115213049	YWHAE	9	5	20
112.	A0A6P7T656	Aconitate hydratase, mitochondrial OS=*Octopus vulgaris*	LOC115219777	ACO1	3	3	5
113.	Cluster-58773.193716	Hypothetical protein LOTGIDRAFT_233221 OS=*Lottia gigantea*	LOTGIDRAFT_233221	LOTGIDRAFT_233221	4	4	11
114.	A0A6P7T402	Uncharacterized HIT-like protein Synpcc7942_1390 OS=*Octopus vulgaris*	LOC115219148	Synpcc7942_1390	6	4	43
115.	Cluster-58773.197059	Hypothetical protein BRAFLDRAFT_281736 OS=*Branchiostoma floridae*	N/A	BRAFLDRAFT_281736	6	1	9
116.	A0A6P7SH66	Alpha-actinin, sarcomeric isoform X3 OS=*Octopus vulgaris*	LOC115212896	actn1	4	4	6
117.	A0A7E6EUH0	Crystallin J1A OS=*Octopus vulgaris*	LOC115212045	CRJ1A	5	3	11
118.	A0A0L8GLX8	S-formylglutathione hydrolase OS=*Octopus bimaculoides*	OCBIM_22031609mg	ESD	4	4	19
119.	A0A0L8HN06	Tubulin alpha chain OS=*Octopus bimaculoides*	OCBIM_22010928mg	TUBA	4	1	12
120.	A0A6P7SEM8	Alpha-mannosidase OS=*Octopus vulgaris*	LOC115211875	MAN2A1	5	5	6
121.	Cluster-58773.192724	PREDICTED: Thioredoxin-like OS=*Amphimedon queenslandica*	LOC100634844	TXNL4A	5	2	23
122.	A0A6P7SUU8	2-iminobutanoate/2-iminopropanoate deaminase OS=*Octopus vulgaris*	LOC115216465	RIDA	8	5	39
123.	A0A6P7SGA6	Clathrin heavy chain OS=*Octopus vulgaris*	LOC115212844	CLTC	3	2	2
124.	A0A0L8GZP6	IF rod domain-containing protein OS=*Octopus bimaculoides*	OCBIM_22025456mg	OCBIM_22025456mg	2	1	7
125.	A0A0L8IEX1	Uncharacterized protein OS=*Octopus bimaculoides*	OCBIM_22014847mg	OCBIM_22014847mg	5	4	23
126.	A0A6P7T0X7	Ganglioside GM2 activator OS=*Octopus vulgaris*	LOC115218607	GM2A	5	3	17
127.	A0A6P7T7L0	Heat shock cognate 71 kDa protein OS=*Octopus vulgaris*	LOC115220772	HSPA8	5	1	8
128.	A0A7E6ENX1	Spectrin alpha chain isoform X2 OS=*Octopus vulgaris*	LOC115227715	Sptan1	3	3	2
129.	Cluster-58773.160867	Soma ferritin OS=*Lymnaea stagnalis*	GenBank: AAB24081.1	FRIS	4	3	20
130.	Cluster-58773.198809	PREDICTED: Carbonic anhydrase 1-like OS=*Aplysia californica*	LOC106013759	CA1	4	3	14
131.	A0A6P7SCY0	Uncharacterized protein OS=*Octopus vulgaris*	LOC115211594		7	4	38
132.	A0A6P7TMY2	Fumarylacetoacetase OS=*Octopus vulgaris*	LOC115226547	Fah	6	1	11
133.	A0A6P7TG90	Glutathione transferase OS=*Octopus vulgaris*	LOC115223852	GST	4	4	19

134.	A0A7E6EK24	Deoxynucleoside triphosphate triphosphohydrolase SAMHD1-like OS=*Octopus vulgaris*	LOC115231394	Samhd1	6	3	11
135.	A0A6P7T9Z5	Thymosin beta isoform X2 OS=*Octopus vulgaris*	LOC115221752	tmsb	3	1	28
136.	A0A6P7T046	Glyoxylate reductase/hydroxypyruvate reductase OS=*Octopus vulgaris*	LOC115218137	grhpr	3	3	15
137.	A0A0L8G0V9	Uncharacterized protein OS=*Octopus bimaculoides*	OCBIM_22003270mg	OCBIM_22003270mg	5	3	7
138.	Cluster-58773.162300	Hemocyanin A-type OS=*Enteroctopus dofleini*	HA	HA	3	1	53
139.	Cluster-58773.169829	Uncharacterized protein LOC105329712 isoform X1 OS=*Crassostrea gigas*	LOC105329712		3	1	25
140.	Cluster-58773.179191	Hypothetical protein BRAFLDRAFT_280591 OS=*Branchiostoma floridae*	N/A	BRAFLDRAFT_280591	2	2	7
141.	Cluster-58773.155745	Hypothetical protein CAPTEDRAFT_117881, partial OS=*Capitella teleta*	CAPTEDRAFT_117881	CAPTEDRAFT_117881	4	2	11
142.	A0A812DBA1	RP-S27Ae OS=*Sepia pharaonis*	SPHA_48671	RP-S27Ae	12	3	24
143.	A0A6P7U923	Fatty acid-binding protein homolog 5 OS=*Octopus vulgaris*	LOC115232159	lbp-5	4	3	31
144.	A0A6P7S7L1	Inositol-1-monophosphatase OS=*Octopus vulgaris*	LOC115209728	suhB	4	3	20
145.	Cluster-58773.187542	PREDICTED: Uncharacterized protein OS=*Aplysia californica*	LOC101849508		3	3	15
146.	A0A6P7T0G1	Phosphoglycerate kinase OS=*Octopus vulgaris*	LOC115218470	pgk	5	4	13
147.	A0A6P7SWF0	Probable methylmalonate-semialdehyde dehydrogenase [acylating], mitochondrial OS=*Octopus vulgaris*	LOC115217127	alh-8	3	3	8
148.	A0A6P7TFF7	Uncharacterized protein OS=*Octopus vulgaris*	LOC115223512		5	3	28
149.	A0A6P7SDM4	Lysosomal aspartic protease isoform X2 OS=*Octopus vulgaris*	LOC115211653		2	2	8
150.	A0A6P7T444	Transforming growth factor-beta-induced protein ig-h3-like OS=*Octopus vulgaris*	LOC115219504	TGFBI	4	3	14
151.	A0A6P7TFF2	Regucalcin OS=*Octopus vulgaris*	LOC115222191	Rgn	4	2	10
152.	A0A6P7SA56	Cathepsin B OS=*Octopus vulgaris*	LOC115210644	Ctsb	5	2	9
153.	A0A6P7S5H1	Mannose-6-phosphate isomerase OS=*Octopus vulgaris*	LOC115209304	MPI	3	2	5
154.	Cluster-58773.152550	Squidulin OS=*Doryteuthis pealeii;* Calcium-binding protein OS=*Watasenia scintillans*	LOC115227564	CABO	3	2	17
155.	A0A6P7TM22	Tetratricopeptide repeat protein 38 OS=*Octopus vulgaris*	LOC115223987	Ttc38	4	4	13
156.	A0A6P7SDZ2	40S ribosomal protein S14 OS=*Octopus vulgaris*	LOC115211938	RPS14	2	2	16
157.	Cluster-58773.108885	Hephaestin-like protein OS=*Acropora millepora*	LOC114955653	HEPHL1	3	3	3
158.	Cluster-1359.1	Tropomyosin OS=*Jasus lalandii*	GenBank: JX860677.1	TM	4	4	14
159.	A0A6P7SVJ8	Arginase OS=*Octopus vulgaris*	LOC115216860	ARG1	2	2	9
160.	A0A6P7SL43	Glucosamine-6-phosphate isomerase OS=*Octopus vulgaris*	LOC115214100	Gnpda1	4	4	17
161.	A0A7E6EXQ9	Cytosolic non-specific dipeptidase isoform X2 OS=*Octopus vulgaris*	LOC115213823	CNDP2	3	1	6
162.	A0A7E6EUU3	Apoptosis-inducing factor 3 isoform X1 OS=*Octopus vulgaris*	LOC115213836	AIFM3	2	2	4
163.	A0A7E6FHK3	Thioredoxin isoform X2 OS=*Octopus vulgaris*	LOC115222284		3	2	28
164.	Cluster-58773.217011	PREDICTED: Prostaglandin reductase 1-like OS=*Crassostrea gigas*	LOC117682985	Ptgr1	3	3	11
165.	A0A6P7TFQ1	Tetraspanin OS=*Octopus vulgaris*	LOC115222869	TSPAN1	6	3	10
166.	A0A0L8G0I6	14_3_3 domain-containing protein OS=*Octopus bimaculoides*	OCBIM_22003454mg	YWHAE	2	2	11

167.	A0A0L8H0K9	Proteasome subunit alpha type OS=*Octopus bimaculoides*	OCBIM_22024847mg	PSMA	2	2	9
168.	A0A7E6FAL0	Protein hu-li tai shao isoform X12 OS=*Octopus vulgaris*	LOC115218883	hts	1	1	2
169.	A0A6P7SK96	Troponin C isoform X2 OS=*Octopus vulgaris*	LOC115213894	TNNC2	2	2	11
170.	A0A6P7U4P1	Deoxynucleoside triphosphate triphosphohydrolase SAMHD1-like isoform X2 OS=*Octopus vulgaris*	LOC115231345	Samhd1	3	3	7
171.	A0A6P7SAF9	Proteasome subunit alpha type OS=*Octopus vulgaris*	LOC115210708	PSMA	4	3	15
172.	A0A7E6ESL5	Prominin-1-A isoform X4 OS=*Octopus vulgaris*	LOC115210705	prom1a	3	3	4
173.	A0A6P7TFA3	Polyol dehydrogenase OS=*Octopus vulgaris*	LOC115223465	Sord	2	2	7
174.	A0A6P7U2G4	Uncharacterized protein LOC115232424 OS=*Octopus vulgaris*	LOC115232424		4	2	4
175.	A0A6P7U2Q2	Uncharacterized protein OS=*Octopus vulgaris*	LOC115232460		2	2	10
176.	A0A7E6EHE9	Prosaposin isoform X1 OS=*Octopus vulgaris*	LOC115209891	Psap	8	4	6
177.	Cluster-58773.136028	PREDICTED: UPF0462 protein C4orf33 homolog OS=*Saccoglossus kowalevskii*	LOC100374324	C4orf33	3	3	12
178.	A0A7E6FG45	Filamin-A isoform X3 OS=*Octopus vulgaris*	LOC115220426	FLNA	2	2	3
179.	A0A812E3Y7	RAB11A OS=*Sepia pharaonis*	SPHA_67892	RAB11A	3	3	12
180.	A0A6P7SDW4	Tropomyosin Tod p 1.0102 OS=*Octopus vulgaris*	LOC115211717	ov-tm	2	2	14
181.	A0A6P7TEG5	Alpha-L-fucosidase OS=*Octopus vulgaris*	LOC115223232	Fuca2	4	3	7
182.	A0A6P7TDL1	Proteasome subunit alpha type OS=*Octopus vulgaris*	LOC115222353	PSMA	5	4	17
183.	A0A0L8HS39	Uncharacterized protein OS=*Octopus bimaculoides*	OCBIM_22007563mg	OCBIM_22007563mg	1	1	18
184.	A0A6P7TQA6	Parkinson disease protein 7 homolog OS=*Octopus vulgaris*	LOC115224773	Park7	2	2	18
185.	A0A6P7SVY8	3-ketoacyl-CoA thiolase, mitochondrial isoform X2 OS=*Octopus vulgaris*	LOC115217007	Acaa2	2	2	7
186.	A0A6P7T033	T-complex protein 1 subunit gamma OS=*Octopus vulgaris*	LOC115218091	Cct3	3	3	7
187.	A0A6P7S854	Neuroglian isoform X1 OS=*Octopus vulgaris*	LOC115210201	Nrg	1	1	2
188.	A0A6P7SYF5	Retinal dehydrogenase 2 OS=*Octopus vulgaris*	LOC115217865	aldh1a2	3	3	7
189.	A0A6P7SHI1	Malate dehydrogenase OS=*Octopus vulgaris*	LOC115212957	MDH	4	2	6
190.	A0A6P7T245	Trans-1,2-dihydrobenzene-1,2-diol dehydrogenase isoform X1 OS=*Octopus vulgaris*	LOC115218668	DHDH	4	3	9
191.	A0A0L8GSZ5	Histone H4 OS=*Octopus bimaculoides*	OCBIM_22029078mg	H4	3	3	14
192.	A0A6P7SU25	Glucose-6-phosphate isomerase OS=*Octopus vulgaris*	LOC115216343	GPI	3	3	5
193.	A0A6P7TDN6	4a-hydroxytetrahydrobiopterin dehydratase OS=*Octopus vulgaris*	LOC115221645	PCBD1	3	3	23
194.	Cluster-58773.132040	Cathepsin L2 OS=*Sinonovacula constricta*	GenBank: KC874990.1	CTSV	2	1	12
195.	A0A6P7SW31	Proteasome subunit alpha type OS=*Octopus vulgaris*	LOC115217043	PSMA	3	3	14
196.	A0A0L8G5K6	Uncharacterized protein OS=*Octopus bimaculoides*	OCBIM_22000375mg	OCBIM_22000375mg	1	1	2
197.	A0A0L8GWF6	Uncharacterized protein OS=*Octopus bimaculoides*	OCBIM_22027193mg	OCBIM_22027193mg	2	1	23
198.	A0A0L8HFB4	Pacifastin domain-containing protein OS=*Octopus bimaculoides*	OCBIM_22015839mg	OCBIM_22015839mg	2	1	5
199.	A0A7E6FJ41	70 kDa Neurofilament protein isoform X2 OS=*Octopus vulgaris*	LOC115222954	Nefl	1	1	3

200.	Cluster-58773.190283	Deoxynucleoside triphosphate triphosphohydrolase SAMHD1-like OS=*Poecilia formosa*	LOC103132374	Samhd1	3	2	8
201.	A0A6P7SLL4	Glutathione S-transferase omega OS=*Octopus vulgaris*	LOC115214295	GSTO1	3	3	14
202.	A0A6P7SRT8	ATP synthase subunit beta OS=*Octopus vulgaris*	LOC115215584	atpb	1	1	3
203.	A0A6P7T526	Probable phosphoglycerate mutase OS=*Octopus vulgaris*	LOC115219769	gpmB	2	2	8
204.	A0A812CP82	Aldehyde dehydrogenase (NAD (+)) OS=*Sepia pharaonis*	SPHA_37438	aldh	3	1	6
205.	A0A0L8HU60	Tetratricopeptide repeat protein 38 OS=*Octopus bimaculoides*	OCBIM_22006847mg	Ttc38	2	2	6
206.	A0A7E6EU42	Rho GDP-dissociation inhibitor 1 isoform X1 OS=*Octopus vulgaris*	LOC115211966	Arhgdia	1	1	8
207.	A0A7E6F650	Ribokinase OS=*Octopus vulgaris*	LOC115217290	rbsK	1	1	7
208.	Cluster-58773.130155	PREDICTED: Mucin-3A-like OS=*Aplysia californica*	LOC115229619	MUC3A	2	2	7
209.	A0A6P7TD81	Dipeptidyl peptidase 2 isoform X1 OS=*Octopus vulgaris*	LOC115222860	DPP7	2	2	6
210.	A0A6P7SCL7	Cylicin-1 OS=*Octopus vulgaris*	LOC115211645	CYLC1	2	2	21
211.	A0A6P7TUU5	Sodium/calcium exchanger regulatory protein 1 OS=*Octopus vulgaris*	LOC115226228		1	1	9
212.	Cluster-58773.109972	Inter-alpha-trypsin inhibitor heavy chain H3-like OS=*Crassostrea gigas*	LOC117683163	ITIH3	4	3	3
213.	A0A0L8H1Z7	CN Hydrolase domain-containing protein OS=*Octopus bimaculoides*	OCBIM_22024094mg	VNN1	2	2	10
214.	Cluster-58773.156686	PREDICTED: Serine hydroxymethyltransferase, cytosolic-like OS=*Crassostrea gigas*	LOC115220966	SHMT1	2	2	4
215.	A0A7E6FKU8	1,2-dihydroxy-3-keto-5-methylthiopentene dioxygenase OS=*Octopus vulgaris*	LOC115223255	mtnD	1	1	11
216.	A0A812CG60	ARF1_2 OS=*Sepia pharaonis*	SPHA_38611	ARF1_2	2	2	13
217.	A0A6P7TV81	Proteasome subunit beta OS=*Octopus vulgaris*	LOC115225870	PSMB	2	2	9
218.	A0A812DGV9	AIFM3 OS=*Sepia pharaonis*	SPHA_53448	AIFM3	1	1	3
219.	A0A6P7SVS2	UV excision repair protein RAD23 OS=*Octopus vulgaris*	LOC115216665	RAD23A	1	1	4
220.	A0A7E6FR50	NADH-cytochrome b5 reductase OS=*Octopus vulgaris*	LOC115224390	CBR1	2	2	5
221.	Cluster-58773.105280	Beta arrestin OS=*Idiosepius paradoxus*	GenBank: LC021440.1	ARRB1	1	1	3
222.	Cluster-58773.201330	17beta-HSD14 OS=*Azumapecten farreri*	GenBank: KC661070.1	HSD17B14	2	2	16
223.	A0A0L8H4W4	Proteasome subunit alpha type OS=*Octopus bimaculoides*	OCBIM_22022293mg	PSMA	1	1	6
224.	A0A6P7T4Z8	Opine dehydrogenase OS=*Octopus vulgaris*	LOC115219799	odh	2	2	7
225.	A0A0L8HB35	CCT-beta OS=*Octopus bimaculoides*	OCBIM_22018710mg	CCT2	1	1	2
226.	Cluster-119530.0	PREDICTED: Tubulin alpha-1B chain isoform X5 OS=*Mandrillus leucophaeus*	LOC105544901	TUBA1B	2	1	29
227.	A0A6P7SLJ4	Alpha-galactosidase OS=*Octopus vulgaris*	LOC115213936	OCBIM_22022443mg	2	2	4
228.	Cluster-58773.109971	PREDICTED: Inter-alpha-trypsin inhibitor heavy chain H3-like OS=*Crassostrea gigas*	LOC117683163	ITIH3	2	2	7
229.	A0A6P7SP60	Peroxiredoxin-2 OS=*Octopus vulgaris*	LOC115215062	Prdx2	1	1	6
230.	A0A6P7SDF8	Pyridoxal 5'-phosphate synthase subunit SNZERR OS=*Octopus vulgaris*	LOC115211795	SNZERR	1	1	4
231.	A0A6P7U678	Collagen alpha-4(VI) chain OS=*Octopus vulgaris*	LOC115232146	COL4A4	1	1	2
232.	A0A6P7SYD8	Neutral protease isoform X1 OS=*Octopus vulgaris*	LOC115217300		1	1	2
233.	A0A0L8FKC8	26S proteasome regulatory subunit 7 OS=*Octopus bimaculoides*	OCBIM_22017212mg	PSMC2	1	1	3

234.	A0A6P7SYN9	Barrier-to-autointegration factor B OS=*Octopus vulgaris*	LOC115217368	banf1-b	1	1	13
235.	A0A7E6FIQ7	Uncharacterized protein isoform X1 OS=*Octopus vulgaris*	LOC115221679		4	2	5
236.	Cluster-58773.152800	PREDICTED: Glutathione S-transferase A-like OS=*Crassostrea gigas*	LOC115210679	GST-T	1	1	4
237.	A0A6P7ST76	Elongation factor 1-beta OS=*Octopus vulgaris*	LOC115216350	EEF1B	1	1	6
238.	A0A7E6ETD0	Adenylyl cyclase-associated protein 1 isoform X4 OS=*Octopus vulgaris*	LOC115210264	Cap1	1	1	2
239.	A0A6P7TBG1	Uridine phosphorylase 1 OS=*Octopus vulgaris*	LOC115220972	Upp1	2	2	14
240.	A0A0L8GUX3	Uncharacterized protein OS=*Octopus bimaculoides*	OCBIM_22027429mg	OCBIM_22027429mg	1	1	3
241.	A0A6P7SYQ3	Pancreatic triacylglycerol lipase-like OS=*Octopus vulgaris*	LOC115217380	Pnlip	1	1	4
242.	A0A6P7SCB7	Gelsolin-like protein 2 OS=*Octopus vulgaris*	LOC115211599	gelsolin	1	1	4
243.	A0A7E6F1S6	Microtubule-associated protein futsch isoform X4 OS=*Octopus vulgaris*	LOC115215514	futsch	1	1	1
244.	A0A6P7TS50	Glutamate--cysteine ligase OS=*Octopus vulgaris*	LOC115225878	GCLC	2	1	2
245.	Cluster-58773.140088	Ecdysteroid-regulated 16 kDa protein OS=*Danaus plexippus*	LOC116770793	ESR16	1	1	5
246.	A0A7E6EZI8	Uncharacterized protein OS=*Octopus vulgaris*	LOC115214248		1	1	0
247.	A0A6P7TK16	Phosphomannomutase OS=*Octopus vulgaris*	LOC115223856	PMM	2	1	6
248.	A0A6P7SE31	26S proteasome non-ATPase regulatory subunit 2 OS=*Octopus vulgaris*	LOC115211842	PSMD2	1	1	1
249.	A0A6P7T1G4	Uncharacterized protein OS=*Octopus vulgaris*	LOC115218491		1	1	2
250.	A0A0L8HJQ8	Peptidase M20 domain-containing protein 2 OS=*Octopus bimaculoides*	OCBIM_22013224mg	PM20D1	1	1	3
251.	A0A7E6ETN4	Protein-glutamine gamma-glutamyltransferase K OS=*Octopus vulgaris*	LOC115211974	TGM1	1	1	2
252.	A0A7E6F497	ATP-citrate synthase OS=*Octopus vulgaris*	LOC115216353	ACLY	1	1	1
253.	Cluster-58773.159728	PREDICTED: Methylmalonic aciduria and homocystinuria type C protein homolog OS=*Saccoglossus kowalevskii*	LOC100368262	Mmachc	1	1	5
254.	A0A7E6EZ05	Vinculin OS=*Octopus vulgaris*	LOC115214348	VCL	1	1	1
255.	A0A6P7S7P4	Peroxiredoxin-5 OS=*Octopus vulgaris*	LOC115209798	Prdx5	1	1	7
256.	A0A812BJI2	Non-specific serine/threonine protein kinase OS=*Sepia pharaonis*	SPHA_16686	STK11	1	1	3
257.	A0A6P7TXK0	Medium-chain specific acyl-CoA dehydrogenase, mitochondrial OS=*Octopus vulgaris*	LOC115228127	Acadm	1	1	4
258.	A0A6P7SUD6	6-phosphogluconolactonase OS=*Octopus vulgaris*	LOC115216558	pgl	1	1	5
259.	A0A6P7TSR4	Carboxypeptidase OS=*Octopus vulgaris*	LOC115226095		1	1	3
260.	A0A0L8G8E4	TGc domain-containing protein OS=*Octopus bimaculoides*	OCBIM_22038334mg	TGM2	1	1	2
261.	Cluster-58773.150174	PREDICTED: Ganglioside GM2 activator-like OS=*Aplysia californica*	LOC115214095	GM2A	2	1	9
262.	A0A6P7S644	Proteasome subunit beta OS=*Octopus vulgaris*	LOC115209401	PSMB	2	1	4
263.	A0A6P7TRV9	Ubiquitin-activating enzyme E1 OS=*Octopus vulgaris*	LOC115225288	UBA1	2	2	2
264.	Cluster-960.0	Sarcoplasmic calcium-binding protein OS=*Artemia franciscana*	SCP1	SCP1	1	1	5
265.	A0A812BBI7	Annexin OS=*Sepia pharaonis*	SPHA_13606	ANXA1	1	1	3
266.	A0A6P7SPL2	Glycogenin-1 isoform X2 OS=*Octopus vulgaris*	LOC115215187	GYG1	1	1	5

267.	A0A6P7TBR4	Golgin subfamily B member 1 isoform X3 OS=*Octopus vulgaris*	LOC115221474	GOLGB1	1	1	2
268.	A0A6P7TPR7	Uncharacterized protein OS=*Octopus vulgaris*	LOC115224696		2	2	3
269.	A0A6P7SKR8	Vesicle-fusing ATPase OS=*Octopus vulgaris*	LOC115213827	NSF	1	1	1
270.	A0A6P7TG43	GDP-4-keto-6-deoxy-D-mannose-3,5-epimerase-4-reductase OS=*Octopus vulgaris*	LOC115222310	fcl	2	2	8
271.	Cluster-58773.163293	PREDICTED: Uncharacterized protein OS=*Aplysia californica*	LOC101860584		2	1	12
272.	A0A6P7T914	Thyroglobulin isoform X2 OS=*Octopus vulgaris*	LOC115220711	TG	2	1	4
273.	A0A0L8ICE8	Calponin-homology (CH) domain-containing protein OS=*Octopus bimaculoides*	OCBIM_22019394mg	SPEF1	1	1	8
274.	A0A6P7T858	Fructose-bisphosphatase OS=*Octopus vulgaris*	LOC115220925	FBP1	2	2	9
275.	A0A7E6FQN6	Insulin-like growth factor-binding protein complex acid labile subunit isoform X7 OS=*Octopus vulgaris*	LOC115225305	Igfals	2	2	4
276.	A0A6P7TUI8	Uncharacterized protein OS=*Octopus vulgaris*	LOC115225721		2	2	4
277.	A0A812DEV9	Rab GDP dissociation inhibitor OS=*Sepia pharaonis*	SPHA_54563	GDI1/GDI2	1	1	2
278.	A0A812CYT6	MYH OS=*Sepia pharaonis*	SPHA_42230	MYH	2	1	1
279.	A0A6P7TEC1	Spermidine synthase OS=*Octopus vulgaris*	LOC115222533	SRM	1	1	4
280.	Cluster-58773.6509	P11221 OPRI_PSEAE Major outer membrane lipoprotein OS=*Pseudomonas aeruginosa* (strain ATCC 15692 / DSM 22644 / CIP 104116 / JCM 14847 / LMG 12228 / 1C / PRS 101 / PAO1)	oprI	oprI	1	1	10
281.	A0A6P7SKQ5	Tropomyosin-2 OS=*Octopus vulgaris*	LOC115213976	TPM2	1	1	6
282.	A0A6P7T124	Cytidine deaminase OS=*Octopus vulgaris*	LOC115218070	CDA	1	1	7
283.	A0A6P7S965	Ras GTPase-activating protein-binding protein 2 OS=*Octopus vulgaris*	LOC115210285	G3BP2	1	1	3
284.	A0A0L8G235	Malate dehydrogenase OS=*Octopus bimaculoides*	OCBIM_22001700mg	MDH	1	1	3
285.	A0A6P7SVM1	Protein-glucosylgalactosylhydroxylysine glucosidase isoform X2 OS=*Octopus vulgaris*	LOC115216968	PGGHG	1	1	2
286.	A0A0L8I519	Sulfotransfer_1 domain-containing protein OS=*Octopus bimaculoides*	OCBIM_22034539mg	DSEL	1	1	3
287.	A0A0L8G0N4	Prolyl endopeptidase OS=*Octopus bimaculoides*	OCBIM_22003476mg	FAP	1	1	2
288.	A0A6P7TDK5	Mucin-5AC-like isoform X1 OS=*Octopus vulgaris*	LOC115222921	MUC5AC	1	1	3
289.	A0A0L8FTG9	Amine oxidase OS=*Octopus bimaculoides*	OCBIM_22008553mg	AOC1	1	1	4
290.	A0A0L8HNM9	Uncharacterized protein (Fragment) OS=*Octopus bimaculoides*	OCBIM_22010506mg	OCBIM_22010506mg	1	1	8
291.	A0A6P7SMT0	Dehydrogenase/reductase SDR family member 4 isoform X1 OS=*Octopus vulgaris*	LOC115214531	DHRS4	1	1	4
292.	A0A812BGH7	Proteasome subunit alpha type OS=*Sepia pharaonis*	SPHA_15160	PSMA	1	1	4
293.	A0A6P7S417	Chloride intracellular channel exc-4 OS=*Octopus vulgaris*	LOC115208761	exc-4	1	1	3
294.	A0A6P7TPC1	Uncharacterized protein isoform X1 OS=*Octopus vulgaris*	LOC115224175		1	1	1
295.	Cluster-58773.214625	Hypothetical protein LOTGIDRAFT_226396 OS=*Lottia gigantea*	LOTGIDRAFT_226396	LOTGIDRAFT_226396	1	1	11
296.	A0A7E6FJ93	Annexin OS=*Octopus vulgaris*	LOC115223011	ANXA1	1	1	2
297.	Q06268	Omega-crystallin OS=*Enteroctopus dofleini*	LOC115217972		1	1	2
298.	A0A7E6F0K3	Phosphonoacetaldehyde hydrolase isoform X2 OS=*Octopus vulgaris*	LOC115215216	phnX	1	1	3

299.	A0A7E6ENY1	Titin-like OS=*Octopus vulgaris*	LOC118762203	TTN	1	1	3
300.	A0A6P7SZG0	cAMP-regulated phosphoprotein 19 OS=*Octopus vulgaris*	LOC115217970	arpp19	1	1	13
301.	A0A7E6EY56	Receptor expression-enhancing protein OS=*Octopus vulgaris*	LOC115213373	REEP1	1	1	4
302.	A0A6P7T976	H(+)-transporting two-sector ATPase OS=*Octopus vulgaris*	LOC115221352	ATP6V1A	1	1	2
303.	A0A0L8GC35	N-acyl-L-amino-acid amidohydrolase OS=*Octopus bimaculoides*	OCBIM_22036825mg	ACY1	1	1	3
304.	A0A6P7TDD8	Guanine nucleotide-binding protein subunit beta-like protein OS=*Octopus vulgaris*	LOC115222872	gnb1l	1	1	5
305.	A0A0L8GMT0	PKS_ER domain-containing protein OS=*Octopus bimaculoides*	OCBIM_22031489mg	OCBIM_22031489mg	1	1	4
306.	A0A6P7SUN7	Hydroxyacylglutathione hydrolase, mitochondrial isoform X1 OS=*Octopus vulgaris*	LOC115216426	hagh	1	1	5
307.	A0A6P7TES8	Uncharacterized protein OS=*Octopus vulgaris*	LOC115222385		1	1	8
308.	A0A0L8G659	IF rod domain-containing protein OS=*Octopus bimaculoides*	OCBIM_22000068mg	OCBIM_22000068mg	1	1	1
309.	A0A7E6FLF6	Leukotriene A-4 hydrolase-like OS=*Octopus vulgaris*	LOC115223638	lkhA	1	1	2
310.	A0A0L8GSS4	AAA domain-containing protein OS=*Octopus bimaculoides*	OCBIM_22029167mg	OCBIM_22029167mg	1	1	3
311.	A0A6P7T7I9	60S ribosomal protein L17 OS=*Octopus vulgaris*	LOC115220375	RPL17	1	1	5
312.	A0A0L8GW99	Uncharacterized protein OS=*Octopus bimaculoides*	OCBIM_22026800mg	OCBIM_22026800mg	1	1	2
313.	A0A7E6FNE1	Dystrophin isoform X4 OS=*Octopus vulgaris*	LOC115232614	Dmd	2	1	0
314.	Cluster-58773.104829	PREDICTED: Calumenin-like isoform X2 OS=*Aplysia californica*	N/A	CALU	1	1	2
315.	A0A0L8I420	Uncharacterized protein (Fragment) OS=*Octopus bimaculoides*	OCBIM_22037121mg	OCBIM_22037121mg	1	1	6
316.	A0A6P7TN39	Proteasome subunit beta OS=*Octopus vulgaris*	LOC115223857	PSMB	1	1	4
317.	A0A0L8FMS3	VWFC domain-containing protein OS=*Octopus bimaculoides*	OCBIM_22013879mg	VWC2	1	1	2
318.	A0A6P7T9M7	Transgelin OS=*Octopus vulgaris*	LOC115221527	SCP1	1	1	2
319.	A0A0L8HS70	XPGN domain-containing protein OS=*Octopus bimaculoides*	OCBIM_22007405mg	ERCC5	1	1	2
320.	A0A0L8IH79	Uncharacterized protein OS=*Octopus bimaculoides*	OCBIM_22005008mg	OCBIM_22005008mg	1	1	2
321.	A0A6P7SJ72	Glutamine--fructose-6-phosphate transaminase (isomerizing) OS=*Octopus vulgaris*	LOC115213781	GFPT1	1	1	2
322.	A0A6P7UBL9	Fatty-acid amide hydrolase 2 isoform X2 OS=*Octopus vulgaris*	LOC115232529	FAAH2	1	1	2
323.	A0A6P7TV68	26S proteasome non-ATPase regulatory subunit 6 OS=*Octopus vulgaris*	LOC115229467	PSMD6	1	1	3
324.	Cluster-58773.94617	E3 ubiquitin-protein ligase TRAF7 OS=*Crassostrea gigas*	LOC105328601	TRAF7	1	1	3
325.	A0A7E6EL05	Uncharacterized protein isoform X1 OS=*Octopus vulgaris*	LOC115232539		1	1	16
326.	Cluster-116787.2	Hypothetical protein NEMVEDRAFT_v1g225725 OS= *Nematostella vectensis*	GenBank: GAMZ01000001.1	NEMVEDRAFT_v1g225725	1	1	2
327.	A0A7E6ENU9	Tumor protein D52 isoform X12 OS=*Octopus vulgaris*	LOC115209283	TPD52	1	1	4
328.	A0A6P7U8T5	Histone H2B OS=*Octopus vulgaris*	LOC115231513	HTB1	1	1	7
329.	A0A6P7TGU1	Actin-interacting protein 1 isoform X1 OS=*Octopus vulgaris*	LOC115223147	AIP1	1	1	1
330.	A0A6P7SB76	Epidermal retinol dehydrogenase 2 isoform X1 OS=*Octopus vulgaris*	LOC115210961	SDR16C5	1	1	5
331.	A0A7E6FD92	Insulin-degrading enzyme OS=*Octopus vulgaris*	LOC115219912	IDE	1	1	1

332.	A0A0L8FU30	Uncharacterized protein OS=*Octopus bimaculoides*	OCBIM_22007941mg	OCBIM_22007941mg	1	1	7
333.	A0A6P7SQ23	Reticulocalbin-2 isoform X2 OS=*Octopus vulgaris*	LOC115215179	RCN2	1	1	3
334.	A0A812BQ89	Transaldolase OS=*Sepia pharaonis*	SPHA_21947	TALDO1	1	1	3
335.	A0A6P7SBT2	Hydroxyacid-oxoacid transhydrogenase OS=*Octopus vulgaris*	LOC115210878	ADHFE1	1	1	2
336.	A0A0L8HXN5	SGL domain-containing protein OS=*Octopus bimaculoides*	OCBIM_22003174mg	OCBIM_22003174mg	1	1	5
337.	A0A0L8GIJ0	VWFA domain-containing protein (Fragment) OS=*Octopus bimaculoides*	OCBIM_22032938mg	OCBIM_22032938mg	1	1	3
338.	A0A6P7TAQ3	Von Willebrand factor C domain-containing protein 2-like OS=*Octopus vulgaris*	LOC115222123	VWC2L	1	1	10
339.	A0A6P7SXS2	Cell surface glycoprotein 1 isoform X6 OS=*Octopus vulgaris*	LOC115217577	env	1	1	1
340.	A0A0L8IEQ2	T-complex protein 1 subunit delta OS=*Octopus bimaculoides*	OCBIM_22015514mg	Cct4	1	1	2
341.	Cluster-86046.0	14-3-3-like protein OS=*Physarum polycephalum*	LOC115229933	fttB	2	1	3
342.	Cluster-58773.110379	AGAP011952-PA OS=*Anopheles gambiae* str. PEST	CASPS3	CASPS3	1	1	4
343.	A0A6P7S7T5	Acid ceramidase-like OS=*Octopus vulgaris*	LOC115209837	ASAH1	1	1	2
344.	A0A812CTD3	Hypothetical protein OS=*Sepia pharaoni*s	SPHA_40360	ALDH1	2	1	4
345.	A0A6P7SZ60	Aldehyde dehydrogenase, mitochondrial OS=*Octopus vulgaris*	LOC115217872	ALDH2	1	1	3
346.	A0A6P7STJ4	Carboxylic ester hydrolase OS=*Octopus vulgaris*	LOC115216415	ACHE	1	1	3
347.	Q24925	Peroxidase-like protein (Fragment) OS=*Euprymna scolopes*	LOC115221592		1	1	2
348.	A0A812DRM9	Hypothetical protein OS=*Sepia pharaonis*	SPHA_58637		1	1	3
349.	Cluster-141421.4	Hypothetical protein ACA1_115170 OS=*Acanthamoeba castellanii* str. Neff	GenBank: KB007926.1	ACA1_115170	1	1	3
350.	A0A0L8GKZ1	Helicase C-terminal domain-containing protein OS=*Octopus bimaculoides*	OCBIM_22031992mg	OCBIM_22031992mg	1	1	1
351.	A0A7E6EGR4	Short-chain collagen C4-like OS=*Octopus vulgaris*	LOC118760927	COL4A4	1	1	4
352.	A0A7E6FUS0	Annexin OS=*Octopus vulgaris*	LOC115226900	ANXA1	1	1	4
353.	A0A812EMG1	DNAH OS=*Sepia pharaonis*	SPHA_78697	DNAH	1	1	1
354.	Q5MX22	Histone H3 (Fragment) OS=*Sepioteuthis lessoniana*	H3	H3	1	1	6
355.	A0A6P7U0P7	Zinc finger ZZ-type and EF-hand domain-containing protein 1 OS=*Octopus vulgaris*	LOC115228010	ZZEF1	1	1	0
356.	A0A0L8IDH2	Non-specific serine/threonine protein kinase OS=*Octopus bimaculoides*	OCBIM_22020713mg	STK11	1	1	2
357.	A0A7E6FDU1	Glucose-6-phosphate 1-dehydrogenase OS=*Octopus vulgaris*	LOC115220357	G6PD	1	1	4
358.	A0A812CC32	Cilia- and flagella-associated protein 65 OS=*Sepia pharaonis*	SPHA_36641	Cfap65	1	1	13
359.	A0A7E6EW14	Tetraspanin OS=*Octopus vulgaris*	LOC115212017	TSPAN1	1	1	5
360.	Cluster-58773.161421	PREDICTED: Troponin I-like OS=*Aplysia californica*	N/A	TNNI1	1	1	4
361.	A0A812DX04	Hypothetical protein OS=*Sepia pharaonis*	SPHA_61893		1	1	3

UPs = Unique Peptides

Cov. [%] = Percentage of Protein Coverage

PSMs = Peptide Spectrum Matches
