# Supplementary material for: Insights into Common Octopus (Octopus vulgaris) Ink Proteome and Bioactive Peptides Using Proteomic Approaches
Source: Mar Drugs. 2023 Mar 24;21(4):206. doi: 10.3390/md21040206 (PMC10142993; doi:10.3390/md21040206)
Supplement: Supplementary file 1 [file marinedrugs-21-00206-s001.zip › Supplementary File S4.pdf]

Supplementary File S4. KEGG pathway analysis of octopus ink proteome by DAVID version 6.8.

No	KEGG Pathway	p-Value	No	KEGG Pathway	p-Value
1	Carbon metabolism	2.20×10^{-12}	12	Biosynthesis of nucleotide sugars	1.10×10^{-02}
2	Biosynthesis of amino acids	7.70×10^{-09}	13	2-Oxocarboxylic acid metabolism	1.40×10^{-02}
3	Fructose and mannose metabolism	1.90×10^{-07}	14	Cysteine and methionine metabolism	2.80×10^{-02}
4	Glycolysis / Gluconeogenesis	1.10×10^{-06}	15	Arginine and proline metabolism	3.30×10^{-02}
5	Metabolic pathways	2.50×10^{-06}	16	beta-Alanine metabolism	3.50×10^{-02}
6	Pentose phosphate pathway	3.60×10^{-05}	17	Fatty acid degradation	4.70×10^{-02}
7	Pyruvate metabolism	3.10×10^{-04}	18	Amino sugar and nucleotide sugar metabolism	5.50×10^{-02}
8	Proteasome	6.90×10^{-04}	19	Arginine biosynthesis	7.00×10^{-02}
9	Drug metabolism - other enzymes	5.00×10^{-03}	20	Pantothenate and CoA biosynthesis	8.70×10^{-02}
10	Citrate cycle (TCA cycle)	9.30×10^{-03}	21	Metabolism of xenobiotics by cytochrome P450	9.80×10^{-02}
11	Glyoxylate and dicarboxylate metabolism	9.30×10^{-03}			
