# Supplementary material for: Insights into Common Octopus (Octopus vulgaris) Ink Proteome and Bioactive Peptides Using Proteomic Approaches
Source: Mar Drugs. 2023 Mar 24;21(4):206. doi: 10.3390/md21040206 (PMC10142993; doi:10.3390/md21040206)
Supplement: Supplementary file 1 [file marinedrugs-21-00206-s001.zip › Supplementary File S5.pdf]

Supplementary File S5. InterPro Motifs analysis of the *O. vulgaris* ink proteome by DAVID version 6.8.

InterPro Motifs	GC	%	p-Value	InterPro Motifs	GC	%	p-Value
GroES-like	4	2.9	2.10×10^{-04}	Tubulin/FtsZ, C-terminal	3	2.2	3.10×10^{-02}
Aldolase-type TIM barrel	4	2.9	3.10×10^{-03}	Calponin homology domain	3	2.2	3.10×10^{-02}
TCP-1-like chaperonin intermediate domain	3	2.2	3.30×10^{-03}	ATPase, alpha/beta subunit, nucleotide-binding domain, active site	2	1.4	3.10×10^{-02}
GroEL-like equatorial domain	3	2.2	3.90×10^{-03}	ATPase, F1/V1/A1 complex, alpha/beta subunit, nucleotide-binding domain	2	1.4	3.10×10^{-02}
GroEL-like apical domain	3	2.2	4.60×10^{-03}	ATPase, alpha/beta subunit, N-terminal	2	1.4	3.10×10^{-02}
Chaperonin Cpn60/TCP-1	3	2.2	4.60×10^{-03}	Tubulin	3	2.2	3.80×10^{-02}
Proteasome, subunit alpha/beta	3	2.2	5.30×10^{-03}	26S proteasome subunit P45	2	1.4	3.90×10^{-02}
ATPase, AAA-type, conserved site	3	2.2	1.30×10^{-02}	AAA+ ATPase domain	4	2.9	4.00×10^{-02}
ATPase, F1 complex beta subunit/V1 complex, C-terminal	2	1.4	1.60×10^{-02}	ATPase, AAA-type, core	3	2.2	4.20×10^{-02}
Lactate/malate dehydrogenase, N-terminal	2	1.4	1.60×10^{-02}	Peptidase M20, dimerization domain	2	1.4	4.70×10^{-02}
L-lactate/malate dehydrogenase	2	1.4	1.60×10^{-02}	Peptidase M20	2	1.4	4.70×10^{-02}
Tubulin, conserved site	3	2.2	1.70×10^{-02}	Proteasome, alpha-subunit, N-terminal domain	2	1.4	4.70×10^{-02}
Glutathione S-transferase, C-terminal	3	2.2	1.80×10^{-02}	Proteasome A-type subunit	2	1.4	5.40×10^{-02}
Glutathione S-transferase, C-terminal-like	3	2.2	2.10×10^{-02}	Alcohol dehydrogenase GroES-like	2	1.4	5.40×10^{-02}
Tubulin, C-terminal	3	2.2	2.20×10^{-02}	SMP-30/Gluconolaconase/LRE-like region	2	1.4	5.40×10^{-02}
Lactate/malate dehydrogenase, C-terminal	2	1.4	2.40×10^{-02}	Thioredoxin, conserved site	2	1.4	6.20×10^{-02}
Glutathione S-transferase, omega-class	2	1.4	2.40×10^{-02}	Alcohol dehydrogenase, C-terminal	2	1.4	6.20×10^{-02}
Glucosamine/galactosamine-6-phosphate isomerase	2	1.4	2.40×10^{-02}	Chaperonin TCP-1, conserved site	2	1.4	6.20×10^{-02}
Smooth muscle protein/calponin	2	1.4	2.40×10^{-02}	Chaperone tailless complex polypeptide 1 (TCP-1)	2	1.4	6.20×10^{-02}
Lactate dehydrogenase/glycoside hydrolase, family 4, C-terminal	2	1.4	2.40×10^{-02}	Pyridoxal phosphate-dependent transferase, major region, subdomain 1	3	2.2	6.20×10^{-02}
Glutathione S-transferase, N-terminal	3	2.2	2.50×10^{-02}	Pyridoxal phosphate-dependent transferase	3	2.2	6.40×10^{-02}
Tubulin/FtsZ, 2-layer sandwich domain	3	2.2	2.50×10^{-02}	Polyketide synthase, enoylreductase	2	1.4	7.70×10^{-02}
Thioredoxin domain	3	2.2	2.70×10^{-02}	Glutamine synthetase/guanido kinase, catalytic domain	2	1.4	8.40×10^{-02}
Tubulin/FtsZ, GTPase domain	3	2.2	2.80×10^{-02}	RmlC-like cupin domain	2	1.4	9.10×10^{-02}

GC: Gene Count.
