# Supplementary material for: Insights into Common Octopus (Octopus vulgaris) Ink Proteome and Bioactive Peptides Using Proteomic Approaches
Source: Mar Drugs. 2023 Mar 24;21(4):206. doi: 10.3390/md21040206 (PMC10142993; doi:10.3390/md21040206)
Supplement: Supplementary file 1 [file marinedrugs-21-00206-s001.zip › Supplementary File S6.pdf]

Supplementary File S6: MCL Clustered Ink Proteins and Descriptions

Clustering Method	*Cluster Number*	*Cluster Color*	*Gene Count*	*Protein Name*	*Protein Identifier*	*Protein Description*
MCL	1	Red	30	Fabp	37653.A0A0L8FWZ8	FABP domain-containing protein
MCL	1	Red	30	OCBIM_22001106mg	37653.A0A0L8G3E3	ATP-citrate synthase; In the C-terminal section; belongs to the succinate/malate CoA ligase alpha subunit family.
MCL	1	Red	30	OCBIM_22001700mg	37653.A0A0L8G235	Malate dehydrogenase
MCL	1	Red	30	OCBIM_22003005mg	37653.A0A0L8G174	1,2-dihydroxy-3-keto-5-methylthiopentene dioxygenase; Catalyzes the formation of formate and 2-keto-4- methylthiobutyrate (KMTB) from 1,2-dihydroxy-3-keto-5-methylthiopentene (DHK-MTPene).
MCL	1	Red	30	OCBIM_22005540mg	37653.A0A0L8FWW9	Glyceraldehyde-3-phosphate dehydrogenase
MCL	1	Red	30	OCBIM_22007559mg	37653.A0A0L8HS44	ATP synthase subunit beta; Produces ATP from ADP in the presence of a proton gradient across the membrane.
MCL	1	Red	30	OCBIM_22007620mg	37653.A0A0L8HTA6	FBPase domain-containing protein; Belongs to the FBPase class 1 family.
MCL	1	Red	30	OCBIM_22009426mg	37653.A0A0L8HPX8	Uncharacterized protein
MCL	1	Red	30	OCBIM_22011519mg	37653.A0A0L8HM12	Uncharacterized protein
MCL	1	Red	30	OCBIM_22012436mg	37653.A0A0L8HKW4	Aconitate hydratase, mitochondrial; Belongs to the aconitase/IPM isomerase family.
MCL	1	Red	30	OCBIM_22013272mg	37653.A0A0L8HKK9	Fructose-bisphosphate aldolase; Belongs to the class I fructose-bisphosphate aldolase family.
MCL	1	Red	30	OCBIM_22014453mg	37653.A0A0L8IDR5	Phosphoglycerate kinase
MCL	1	Red	30	OCBIM_22015943mg	37653.A0A0L8HGI4	6-phosphogluconolactonase; Hydrolysis of 6-phosphogluconolactone to 6-phosphogluconate.
MCL	1	Red	30	OCBIM_22016575mg	37653.A0A0L8HEP1	Aspartate aminotransferase
MCL	1	Red	30	OCBIM_22018431mg	37653.A0A0L8ICW2	Transaldolase; Transaldolase is important for the balance of metabolites in the pentose-phosphate pathway.
MCL	1	Red	30	OCBIM_22020205mg	37653.A0A0L8H8S0	Uncharacterized protein; Belongs to the LDH/MDH superfamily.
MCL	1	Red	30	OCBIM_22022789mg	37653.A0A0L8FFZ3	Tyrosinase_Cu-bd domain-containing protein

Clustering Method	Cluster Number	Cluster Color	Gene Count	Protein Name	Protein Identifier	Protein Description
MCL	1	Red	30	OCBIM_22025350mg	37653.A0A0L8H0R2	Clathrin-link domain-containing protein
MCL	1	Red	30	OCBIM_22026553mg	37653.A0A0L8IA05	Uncharacterized protein; Belongs to the peptidase M18 family.
Clustering Method	*Cluster Number*	*Cluster Color*	*Gene Count*	*Protein Name*	*Protein Identifier*	*Protein Description*
MCL	1	Red	30	OCBIM_22027353mg	37653.A0A0L8GV19	Glucose-6-phosphate 1-dehydrogenase; Catalyzes the rate-limiting step of the oxidative pentose- phosphate pathway, which represents a route for the dissimilation of carbohydrates besides glycolysis.
MCL	1	Red	30	OCBIM_22031231mg	37653.A0A0L8GMM5	Ferritin; Stores iron in a soluble, non-toxic, readily available form. Important for iron homeostasis. Iron is taken up in the ferrous form and deposited as ferric hydroxides after oxidation. ; Belongs to the ferritin family.
MCL	1	Red	30	OCBIM_22031609mg	37653.A0A0L8GLX8	S-formylglutathione hydrolase; Serine hydrolase involved in the detoxification of formaldehyde.
MCL	1	Red	30	OCBIM_22031951mg	37653.A0A0L8GKM6	Nucleoside diphosphate kinase
MCL	1	Red	30	OCBIM_22032473mg	37653.A0A0L8GJH5	Pyruvate kinase; Belongs to the pyruvate kinase family.
MCL	1	Red	30	OCBIM_22035502mg	37653.A0A0L8GDJ1	Uncharacterized protein
MCL	1	Red	30	OCBIM_22037419mg	37653.A0A0L8I2N5	Triosephosphate isomerase
MCL	1	Red	30	OCBIM_22037603mg	37653.A0A0L8G9D6	PKS_ER domain-containing protein
MCL	1	Red	30	OCBIM_22038393mg	37653.A0A0L8G7X1	Uncharacterized protein
MCL	1	Red	30	OCBIM_22039086mg	37653.A0A0L8G7Y6	Catalase domain-containing protein
MCL	1	Red	30	OCBIM_22039213mg	37653.A0A0L8G7T8	Iso_dh domain-containing protein
MCL	2	Brown	18	OCBIM_22005008mg	37653.A0A0L8IH79	Uncharacterized protein; Belongs to the chaperonin (HSP60) family.
MCL	2	Brown	18	OCBIM_22005110mg	37653.A0A0L8IFP8	Superoxide dismutase [Cu-Zn]; Destroys radicals which are normally produced within the cells and which are toxic to biological systems. ; Belongs to the Cu-Zn superoxide dismutase family.
MCL	2	Brown	18	OCBIM_22006581mg	37653.A0A0L8FWK7	Uncharacterized protein; Belongs to the universal ribosomal protein uL22 family.
MCL	2	Brown	18	OCBIM_22013251mg	37653.A0A0L8FNH6	Proteasome subunit beta
MCL	2	Brown	18	OCBIM_22015514mg	37653.A0A0L8IEQ2	T-complex protein 1 subunit delta
MCL	2	Brown	18	OCBIM_22017212mg	37653.A0A0L8FKC8	AAA domain-containing protein; Belongs to the AAA ATPase family.

MCL	2	Brown	18	OCBIM_22018710mg	37653.A0A0L8HB35	Uncharacterized protein
MCL	2	Brown	18	OCBIM_22021443mg	37653.A0A0L8H7N1	UBA domain-containing protein
MCL	2	Brown	18	OCBIM_22022293mg	37653.A0A0L8H4W4	Proteasome subunit alpha type
MCL	2	Brown	18	OCBIM_22024200mg	37653.A0A0L8FF74	AA_TRNA_LIGASE_II domain-containing protein
Clustering Method	***Cluster Number***	***Cluster Color***	***Gene Count***	***Protein Name***	***Protein Identifier***	***Protein Description***
MCL	2	Brown	18	OCBIM_22024847mg	37653.A0A0L8H0K9	Proteasome subunit alpha type
MCL	2	Brown	18	OCBIM_22025440mg	37653.A0A0L8GZ36	Uncharacterized protein
MCL	2	Brown	18	OCBIM_22027429mg	37653.A0A0L8GUX3	Uncharacterized protein; Belongs to the universal ribosomal protein uL3 family.
MCL	2	Brown	18	OCBIM_22029167mg	37653.A0A0L8GSS4	AAA domain-containing protein; Belongs to the AAA ATPase family.
MCL	2	Brown	18	OCBIM_22036340mg	37653.A0A0L8GBS0	PCI domain-containing protein
MCL	2	Brown	18	OCBIM_22036439mg	37653.A0A0L8GBA9	26S proteasome non-ATPase regulatory subunit 2; Component of the 26S proteasome, a multiprotein complex involved in the ATP-dependent degradation of ubiquitinated proteins. This complex plays a key role in the maintenance of protein homeostasis by removing misfolded or damaged proteins, which could impair cellular functions, and by removing proteins whose functions are no longer required. Therefore, the proteasome participates in numerous cellular processes, including cell cycle progression, apoptosis, or DNA damage repair; Belongs to the proteasome subunit S2 family.
MCL	2	Brown	18	OCBIM_22036500mg	37653.A0A0L8GCM1	Uncharacterized protein
MCL	2	Brown	18	OCBIM_22037121mg	37653.A0A0L8I420	Uncharacterized protein
MCL	3	Dark Golden Rod	7	OCBIM_22000521mg	37653.A0A0L8HZY4	Fumarylacetoacetase
MCL	3	Dark Golden Rod	7	OCBIM_22003650mg	37653.A0A0L8FZK6	ADH_zinc_N domain-containing protein
MCL	3	Dark Golden Rod	7	OCBIM_22008553mg	37653.A0A0L8FTG9	Amine oxidase
MCL	3	Dark Golden Rod	7	OCBIM_22008657mg	37653.A0A0L8HQM9	Uncharacterized protein; Belongs to the short-chain dehydrogenases/reductases (SDR) family.

Clustering Method	*Cluster Number*	*Cluster Color*	*Gene Count*	*Protein Name*	*Protein Identifier*	*Protein Description*
MCL	3	Dark Golden Rod	7	OCBIM_22023716mg	37653.A0A0L8FFE9	Uncharacterized protein
MCL	3	Dark Golden Rod	7	OCBIM_22036170mg	37653.A0A0L8GC14	ACPS domain-containing protein
MCL	3	Dark Golden Rod	7	OCBIM_22037270mg	37653.A0A0L8GAD8	Uncharacterized protein; Belongs to the GST superfamily.
MCL	4	Yellow	7	OCBIM_22005448mg	37653.A0A0L8HW02	Uncharacterized protein
MCL	4	Yellow	7	OCBIM_22022828mg	37653.A0A0L8H417	Glucose-6-phosphate isomerase; Belongs to the GPI family.
MCL	4	Yellow	7	OCBIM_22024348mg	37653.A0A0L8H1I1	Xylose isomerase
Clustering Method	*Cluster Number*	*Cluster Color*	*Gene Count*	*Protein Name*	*Protein Identifier*	*Protein Description*
MCL	4	Yellow	7	OCBIM_22026276mg	37653.A0A0L8GXA0	Glucosamine-6-phosphate isomerase
MCL	4	Yellow	7	OCBIM_22028783mg	37653.A0A0L8GS34	Uncharacterized protein
MCL	4	Yellow	7	OCBIM_22032752mg	37653.A0A0L8GJ57	Ribokinase; Catalyzes the phosphorylation of ribose at O-5 in a reaction requiring ATP and magnesium. The resulting D-ribose-5-phosphate can then be used either for sythesis of nucleotides, histidine, and tryptophan, or as a component of the pentose phosphate pathway.
MCL	4	Yellow	7	OCBIM_22037884mg	37653.A0A0L8G8V0	Phosphomannomutase; Involved in the synthesis of the GDP-mannose and dolichol- phosphate-mannose required for a number of critical mannosyl transfer reactions.
MCL	5	Olive	4	OCBIM_22019212mg	37653.A0A0L8HAQ5	Aldedh domain-containing protein; Belongs to the aldehyde dehydrogenase family.
MCL	5	Olive	4	OCBIM_22024094mg	37653.A0A0L8H1Z7	CN hydrolase domain-containing protein
MCL	5	Olive	4	OCBIM_22026800mg	37653.A0A0L8GW99	Uncharacterized protein
MCL	5	Olive	4	OCBIM_22030334mg	37653.A0A0L8GP20	Aldehyde dehydrogenase; Belongs to the aldehyde dehydrogenase family.
MCL	6	Green	4	OCBIM_22000781mg	37653.A0A0L8HZX3	Arginase
MCL	6	Green	4	OCBIM_22019540mg	37653.A0A0L8H9P1	Uncharacterized protein; Belongs to the ATP:guanido phosphotransferase family.
MCL	6	Green	4	OCBIM_22022443mg	37653.A0A0L8H4W2	Alpha-galactosidase
MCL	6	Green	4	OCBIM_22036825mg	37653.A0A0L8GC35	M20_dimer domain-containing protein
MCL	7	Green 2	4	OCBIM_22003270mg	37653.A0A0L8G0V9	Uncharacterized protein; Belongs to the heat shock protein 70 family.

MCL	7	Green 2	4	OCBIM_22026436mg	37653.A0A0L8GWN9	Carboxypeptidase; Belongs to the peptidase S10 family.
MCL	7	Green 2	4	OCBIM_22027793mg	37653.A0A0L8I919	Calreticulin
MCL	7	Green 2	4	OCBIM_22033046mg	37653.A0A0L8GJH1	Uncharacterized protein; Belongs to the protein disulfide isomerase family.
MCL	8	Light Green	3	OCBIM_22003454mg	37653.A0A0L8G0I6	14_3_3 domain-containing protein; Belongs to the 14-3-3 family.
MCL	8	Light Green	3	OCBIM_22009637mg	37653.A0A0L8FRZ6	14_3_3 domain-containing protein; Belongs to the 14-3-3 family.
MCL	8	Light Green	3	OCBIM_22020713mg	37653.A0A0L8IDH2	Protein kinase domain-containing protein; Belongs to the protein kinase superfamily.
MCL	9	Medium Sea Green	3	MYH	37653.A0A0L8H7W7	Uncharacterized protein
MCL	9	Medium Sea Green	3	OCBIM_22005155mg	37653.A0A0L8FYB6	Uncharacterized protein
MCL	9	Medium Sea Green	3	OCBIM_22007563mg	37653.A0A0L8HS39	Uncharacterized protein
Clustering Method	***Cluster Number***	***Cluster Color***	***Gene Count***	***Protein Name***	***Protein Identifier***	***Protein Description***
MCL	10	Cyan	2	OCBIM_22018475mg	37653.A0A0L8FIV0	Uncharacterized protein
MCL	11	Medium Purple	2	OCBIM_22015214mg	37653.A0A0L8FLK6	Cytidine deaminase; This enzyme scavenges exogenous and endogenous cytidine and 2'-deoxycytidine for UMP synthesis; Belongs to the cytidine and deoxycytidylate deaminase family.
MCL	11	Medium Purple	2	OCBIM_22032061mg	37653.A0A0L8GLL0	Thymidine phosphorylase; Catalyzes the reversible phosphorolysis of thymidine. The produced molecules are then utilized as carbon and energy sources or in the rescue of pyrimidine bases for nucleotide synthesis. ; Belongs to the thymidine/pyrimidine-nucleoside phosphorylase family.
MCL	12	Orchid	2	OCBIM_22027999mg	37653.A0A0L8GU71	VOC domain-containing protein
MCL	12	Orchid	2	hagh	37653.A0A0L8GMH9	HAGH_C domain-containing protein
MCL	13	Purple	2	OCBIM_22002474mg	37653.A0A0L8G0U4	Serine hydroxymethyltransferase; Interconversion of serine and glycine. ; Belongs to the SHMT family.
MCL	13	Purple	2	OCBIM_22031492mg	37653.A0A0L8GLQ4	10-formyltetrahydrofolate dehydrogenase; In the C-terminal section; belongs to the aldehyde dehydrogenase family. ALDH1L subfamily.
MCL	14	Hot Pink	2	OCBIM_22002213mg	37653.A0A0L8G196	NAD(P)-bd_dom domain-containing protein

MCL	14	Hot Pink	2	OCBIM_22033873mg	37653.A0A0L8GGM9	Epimerase domain-containing protein
MCL	15	Pink	2	OCBIM_22012269mg	37653.A0A0L8HLB5	Adenosylhomocysteinase
MCL	15	Pink	2	OCBIM_22035593mg	37653.A0A0L8I4B3	Uncharacterized protein
