# Supplementary figures and images for: Insights into Common Octopus (Octopus vulgaris) Ink Proteome and Bioactive Peptides Using Proteomic Approaches
Source: Mar Drugs. 2023 Mar 24;21(4):206. doi: 10.3390/md21040206 (PMC10142993; doi:10.3390/md21040206)

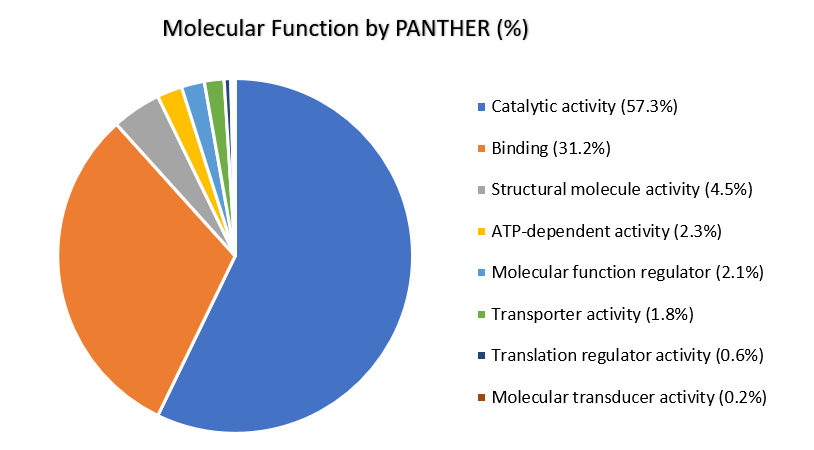

Supplement: Supplementary file 1 [file marinedrugs-21-00206-s001.zip › Supplementary Figure S1.png]

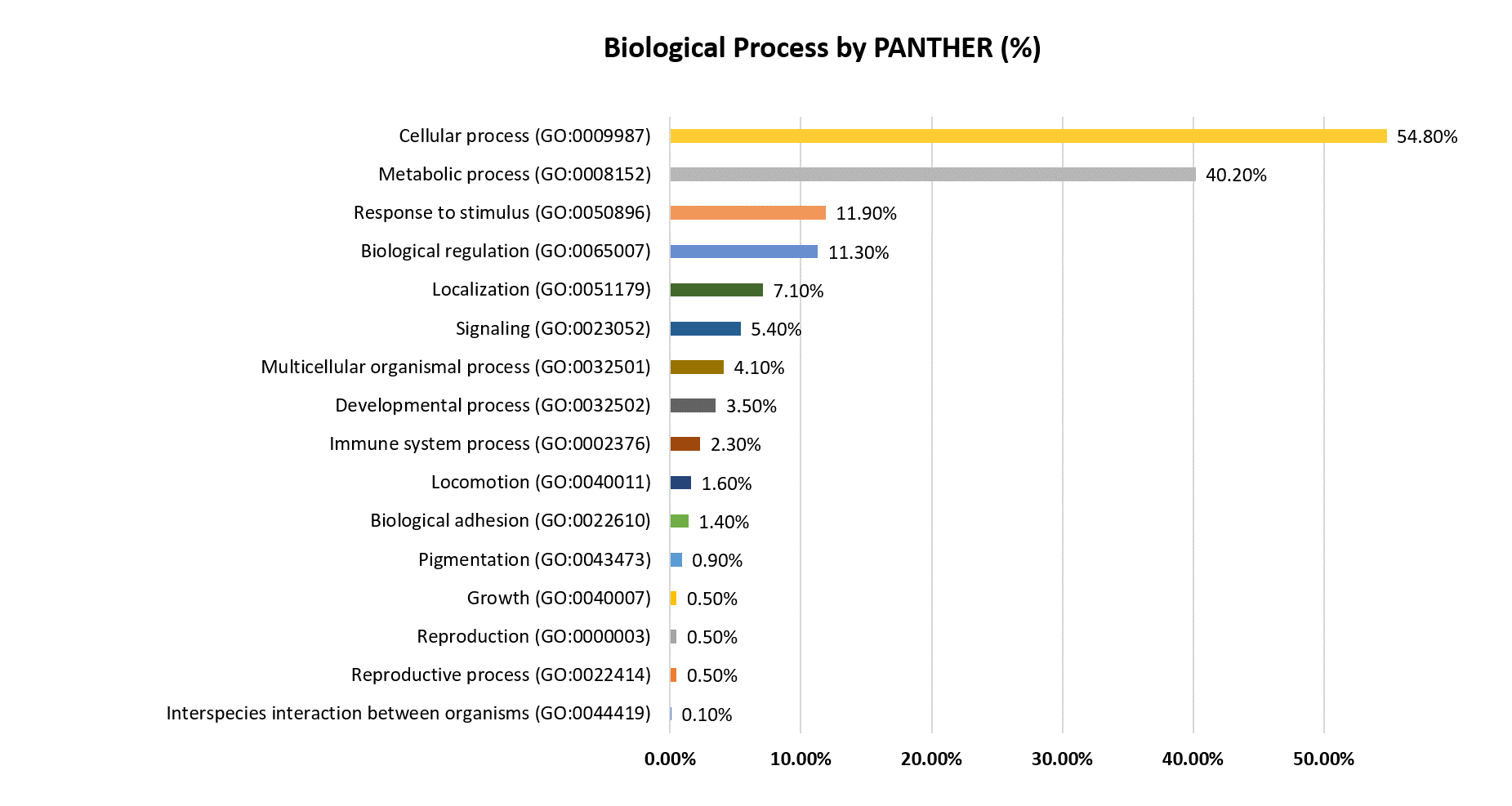

Supplement: Supplementary file 1 [file marinedrugs-21-00206-s001.zip › Supplementary Figure S2.png]
